# A Comprehensive Approach to the Thyroid Bethesda Category III (AUS) in the Transition Zone Between 2nd Edition and 3rd Edition of The Bethesda System for Reporting Thyroid Cytopathology: Subcategorization, Nuclear Scoring, and More

**DOI:** 10.1007/s12022-024-09797-1

**Published:** 2024-01-27

**Authors:** Merve Bagıs, Nuray Can, Necdet Sut, Ebru Tastekin, Ezgi Genc Erdogan, Buket Yilmaz Bulbul, Yavuz Atakan Sezer, Osman Kula, Elif Mercan Demirtas, Inci Usta

**Affiliations:** 1 Department of Pathology, Faculty of Medicine, Trakya University, 22030 Edirne, Turkey; 2 Department of Biostatistics, Faculty of Medicine, Trakya University, 22030 Edirne, Turkey; 3 Division of Endocrinology and Metabolism, Department of Internal Medicine, Faculty of Medicine, Trakya University, 22030 Edirne, Turkey; 4 Department of General Surgery, Faculty of Medicine, Trakya University, 22030 Edirne, Turkey; 5 Department of Radiology, Faculty of Medicine, Trakya University, 22030 Edirne, Turkey; 6 Department of Pathology, Adiyaman University Training and Research Hospital, 02040 Adiyaman, Turkey

**Keywords:** Atypia of undetermined significance, Risk of malignancy, Subcategorization, Nuclear atypia, Nuclear score, The Bethesda System

## Abstract

Significant interobserver variabilities exist for Bethesda category III: atypia of undetermined significance (AUS) of *The Bethesda System for Reporting Thyroid Cytopathology* (TBSRTC). Thus, subcategorization of AUS including AUS “nuclear” and AUS “other” is proposed in the recent 3rd edition of TBSRTC. This study investigated the impact of the nuclear features/architectural features/nuclear score (NS) (3-tiered)/subcategories and subgroups on risk of malignancy (ROM) in thyroid fine-needle aspirations (FNA). 6940 FNAs were evaluated. 1224 (17.6%) cases diagnosed as AUS were reviewed, and 240 patients (initial FNAs of 260 nodules and 240 thyroidectomies) were included. Subcategories and subgroups were defined according to TBSRTC 2nd and 3rd editions. Histological diagnostic groups included nonneoplastic disease, benign neoplasm, low-risk neoplasm, and malignant neoplasm. Overall, ROM was 30.7%. ROM was significantly higher in FNAs with nuclear overlapping (35.5%), nuclear molding (56.9%), irregular contours (42.1%), nuclear grooves (74.1%), chromatin clearing (49.4%), and chromatin margination (57.7%), and these features were independent significant predictors for malignancy. FNAs with NS3 had significantly higher ROM (64.2%). Three-dimensional groups were significantly more frequent in malignant neoplasms (35.7%). ROM was significantly higher in AUS-nuclear subcategory (48.2%) and in AUS-nuclear and architectural subcategory (38.3%). The highest ROM was detected in AUS-nuclear1 subgroup (65.2%). ROM was significantly higher in the group including AUS-nuclear and AUS-nuclear and architectural subcategories, namely “high-risk group” than the group including other subcategories, namely “low-risk group” (42.0%vs 13.9%). In conclusion, subcategorization may not be the end point, and nuclear scoring and evaluation of architectural patterns according to strict criteria may provide data for remodeling of TBSRTC categories.

## Introduction

Since the first study indicating the relationship between abnormal thyroid tissue and increased echogenity with a low-resolution B-Mode ultrasound have been reported by Fujimoto et al. [1] in 1967, the incidence of thyroid nodules detected by ultrasonography has been increased with an epidemic-like trend. Although physical examination enables determining thyroid nodules in only approximately 5 to 7% of the adult population, the frequency of detected thyroid nodules with ultrasonography exceeds 60% of the adult population [2]. More than 90% of thyroid nodules are benign lesions of no clinical significance, but approximately 4 to 6.5% of thyroid nodules may be clinically significant since that they may represent thyroid cancer [2, 3]. Following the first description of fine-needle aspiration (FNA) techniques by Martin and Ellis in 1930 [4] and the recommendation of ultrasound guidance by Walfish et al. in 1977 [5], ultrasonography-guided FNA is the standard tool for the appropriate clinical management of thyroid nodules. Thyroid FNA accompanied by advanced ultrasonographic imaging is a safe, inexpensive, and minimal invasive method in detecting thyroid nodules that require surgery [6]. In this context, *The Bethesda System for Reporting Thyroid Cytopathology* (TBSRTC) is a worldwide accepted reporting system for thyroid FNAs since the first edition reported following National Cancer Institute “Thyroid FNA State of the Science Conference” held in Bethesda, MD, in October of 2007 [7]. TBSRTC was developed to standardize the diagnostic terminology of thyroid cytopathology and consists of six categories, each of which indicates a particular risk of malignancy (ROM) which ensures that patients benefit from appropriate clinical management by using the same language between the pathologist and the surgeon or endocrinologist [6, 8]. However, significant interobserver variabilities exist for indeterminate categories due to the subjective morphological interpretation. Of these categories, undoubtedly one of the most problematic is the Bethesda category III: atypia of undetermined significance (AUS). The recommendation about the upper limit of the AUS diagnostic category to no more than 10% of thyroid FNAs in the 2nd edition of TBSRTC [9] continues unchanged, and also a suggestion about AUS:malignant ratio ≤ 3.0 as a laboratory quality control was included in the recently published TBSRTC 3rd edition [6]. The AUS category has been simplified by removing the term “follicular lesion of undetermined significance (FLUS),” and the possible scenarios presented in the TBSRTC 2nd edition are organized and subdivided into subcategories as “AUS-nuclear” and “AUS-other” in the recent edition of TBSRTC [6, 9]. However, the rates for AUS varying between 1 and 22% among laboratories have been reported in the literature [10]. So, it can be argued that the category with the lowest agreement between cytopathologists is the AUS category according to the studies in the literature [11–14]. The heterogeneity of this category, the subjectivity of interpretation, and the lack of a set of objective criteria, as many believe, lead to the overuse of this category and the decrease in harmony between cytopathologists [10]. In addition, it is difficult to determine the ROM since only a small portion of AUS cases has surgical follow-ups. The predicted mean ROM for AUS category reported in the TBSRTC 3rd edition as 22 (13–30%) [6]. However, the reported risk of histological proved malignancy in the literature varies between 17 and 83%, showing a wide range [15, 16].

Many authors have emphasized subcategorizing AUS category due to their wide range of malignancy risk and the heterogeneous nature of the category, and they have reported higher rates of malignancy in AUS with nuclear or cytological atypia than AUS with architectural atypia [8, 16–27]. And finally, two-tiered subcategorization of AUS including AUS “nuclear” and AUS “other” is recommended in the recent 3rd edition of TBSRTC [6]. While the definition of AUS, nuclear subcategory includes the criteria as focal nuclear atypia, diffuse but mild nuclear atypia, nuclear and architectural atypia, atypical cyst-lining cells, and histiocytoid cells, AUS; other subcategory consists of architectural atypia (defined by three different scenarios); oncocytic atypia (two scenarios); atypia, not otherwise specified (three scenarios); and atypical lymphoid cells [6]. At this point, new questions, which are the subjects of the present study, may emerge as in the following: Why does not every case of nuclear atypia exhibit the same ROM? Are there nuclear features that may predict a higher ROM? Can a more objective approach be obtained by scoring nuclear features in predicting malignancy? Can the ROM differ according to the criteria defined for the subcategories?

So, this study aims to investigate the impact of the subcategories, the impact of the criteria defining these subcategories, and the effects of nuclear and architectural features on risk of malignancy in AUS. And also, the authors aim to investigate the producibility and usefulness of a nuclear scoring system in order to achieve a more objective evaluation of nuclear features in thyroid FNA cytology.

## Material and Methods

### Study Design

**Fig. 1:**
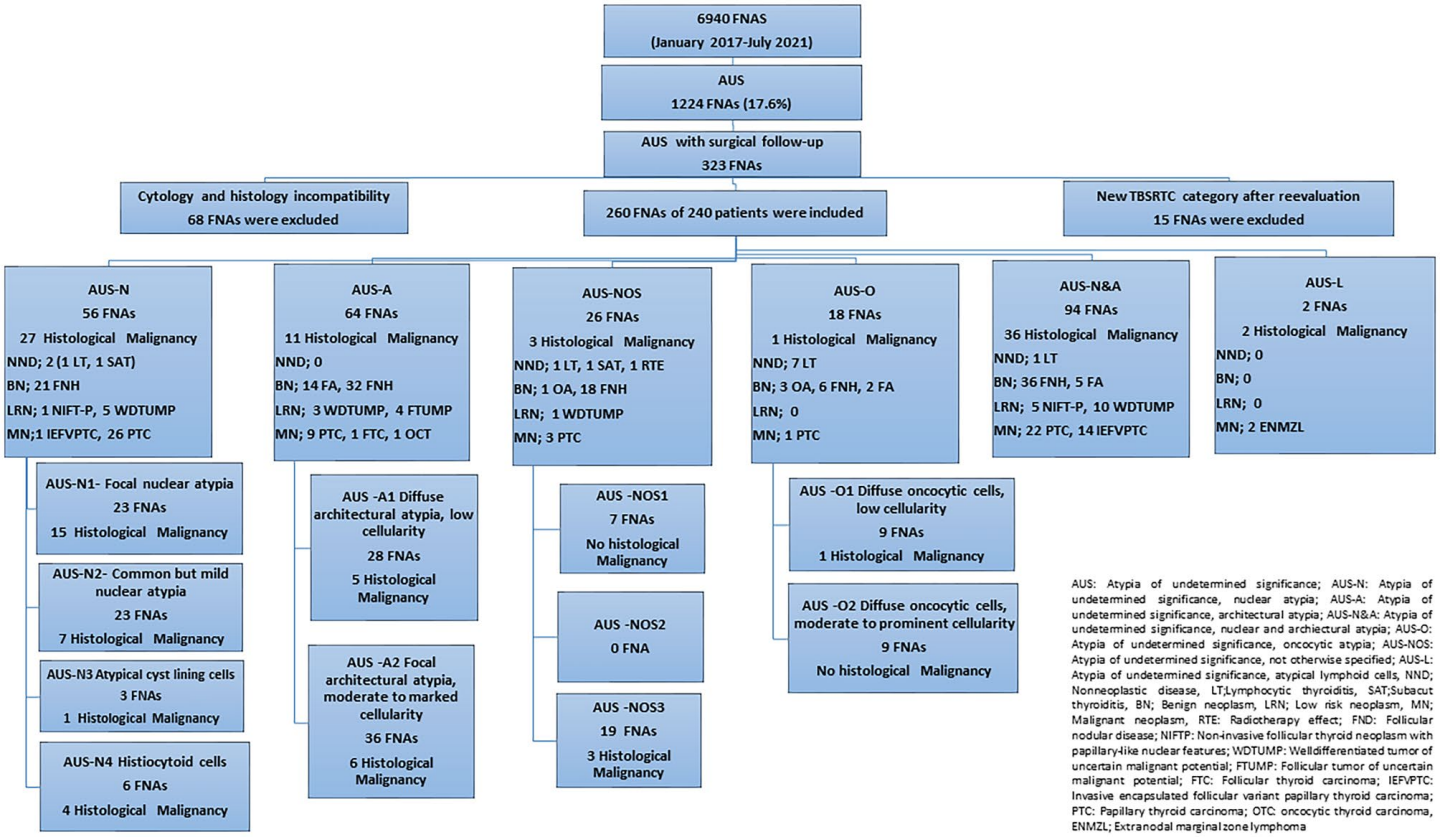
Study design of the present study

The records of 6940 thyroid FNAs evaluated in Department of Pathology between January 2017 and July 2021 were reviewed. The study design was approved by the local Ethic Committee of the University Hospital (Protocol Code: TÜTF-GOBAEK 2023/76). 1224 (17.6%) cases diagnosed with AUS/FLUS according to TSRBS 2nd edition [9] were reevaluated. In order to rule out reactive changes due to the previous aspirations, cases diagnosed with AUS/FLUS in the first aspiration materials of the nodules were included in the study. To ensure histopathological correlation, 323 cases with resection materials (hemithyroidectomy/total thyroidectomy) were evaluated. The compatibility of the aspirated target nodule with the nodule observed in the resection was achieved by using ultrasonographic findings, macroscopic examination of the resection material, and histomorphological changes due to previous FNAs. Sixty-eight cases for whom correlation could not be established (nodules of similar size located close to each other in cases with multiple nodules, nodules whose ultrasonography report could not be obtained, nodules of similar size of which exact location was not specified in the resection macroscopy report, etc.) were excluded from the study. Of the cases that met the criteria, 15 cases were assigned to other TBSRTC categories (Nondiagnostic 1, Benign 13, Follicular neoplasia 1) after a diagnostic revision. As a result, 260 FNAs of 260 nodules and 240 resection materials of 240 patients were included in the study (Fig. 1).

### Histopathological and Cytological Evaluation

In cytological evaluation, May-Grünwald Giemsa (MGG) and Papanicolau (PAP)-stained conventional smears and PAP-stained preparations obtained from the liquid-based cytology (LBS) method were used. Cytological evaluation was performed blindly, devoid of all other information except age, gender, nodule size, and location. The cellularity and colloid status of the aspirates were evaluated. The presence of cytological atypia, nuclear score (NS), and the extent of NS was determined. Architectural patterns and their extent were recorded. The presence of atypical cyst-lining cells, histiocytoid cells, oncocytic cells, psammoma bodies, histiocytes, lymphocytes, and atypical lymphoid cells was evaluated.

Hematoxylin & Eosin (H&E)-stained slides of formalin-fixed paraffin-embedded tissues were used in histopathological evaluation, and the histopathological diagnosis was revised according to the 5th edition of World Health Organization (WHO) Classification of thyroid tumors (Beta version) [28]. The cases were divided into four groups according to their histology: non-neoplastic disease (NND), benign neoplasm (BN), low-risk neoplasm (LRN), and malignant neoplasm (MN) (Table 1, Fig. 1).

**Table 1:** Cinicopathological features of the study group

Parameters			***n*** (%)	Total
Age (mean ± SD)	50.1 ± 11.9 years			
Gender	**Female**		203 (78.1)	260 (100)
	**Male**		57 (21.9)	
Nodule size	** < 1 cm**		47 (18.1)	260 (100)
	** ≥ 1 cm**		213 (81.9)	
Nodule localization	**Left thyroid lobe**		122 (46.9)	260 (100)
	**Isthmus**		6 (2.3)	
	**Right thyroid lobe**		132 (50.8)	
Histological diagnosis	**Nonneoplastic disease**	**Lymphocytic thyroiditis**	10 (76.9)	13 (5)
		**Subacute thyroiditis**	2 (15.4)	
		**Radiotherapy related features**	1 (7.7)	
	**Benign neoplasm**	**Follicular nodular disease**	113 (81.9)	138 (53.1)
		**Follicular adenoma**	21 (15.2)	
		**Oncocytic adenoma**	4 (2.9)	
	**Low-risk neoplasm**	**NIFTP**	6 (20.6)	29 (11.2)
		**FT-UMP**	4 (13.8)	
		**WDT-UMP**	19 (65.6)	
	**Malignant neoplasm**	**Follicular thyroid carcinoma**	1 (1.2)	80 (30.7)
		**Invasive encapsulated follicular variant of papillary thyroid carcinoma**	15 (18.8)	
		**Papillary thyroid carcinoma**	61 (76.3)	
		**Oncocytic carcinoma**	1 (1.2)	
		**Extranodal marginal zone lymphoma**	2 (2.5)	

*NIFTP* non-invasive follicular thyroid neoplasm with papillary-like nuclear features, *WDT-UMP* well-differentiated tumor of uncertain malignant potential, *FT-UMP* follicular tumor of uncertain malignant potential.

#### Evaluation of Nuclear Features

**Fig. 2:**
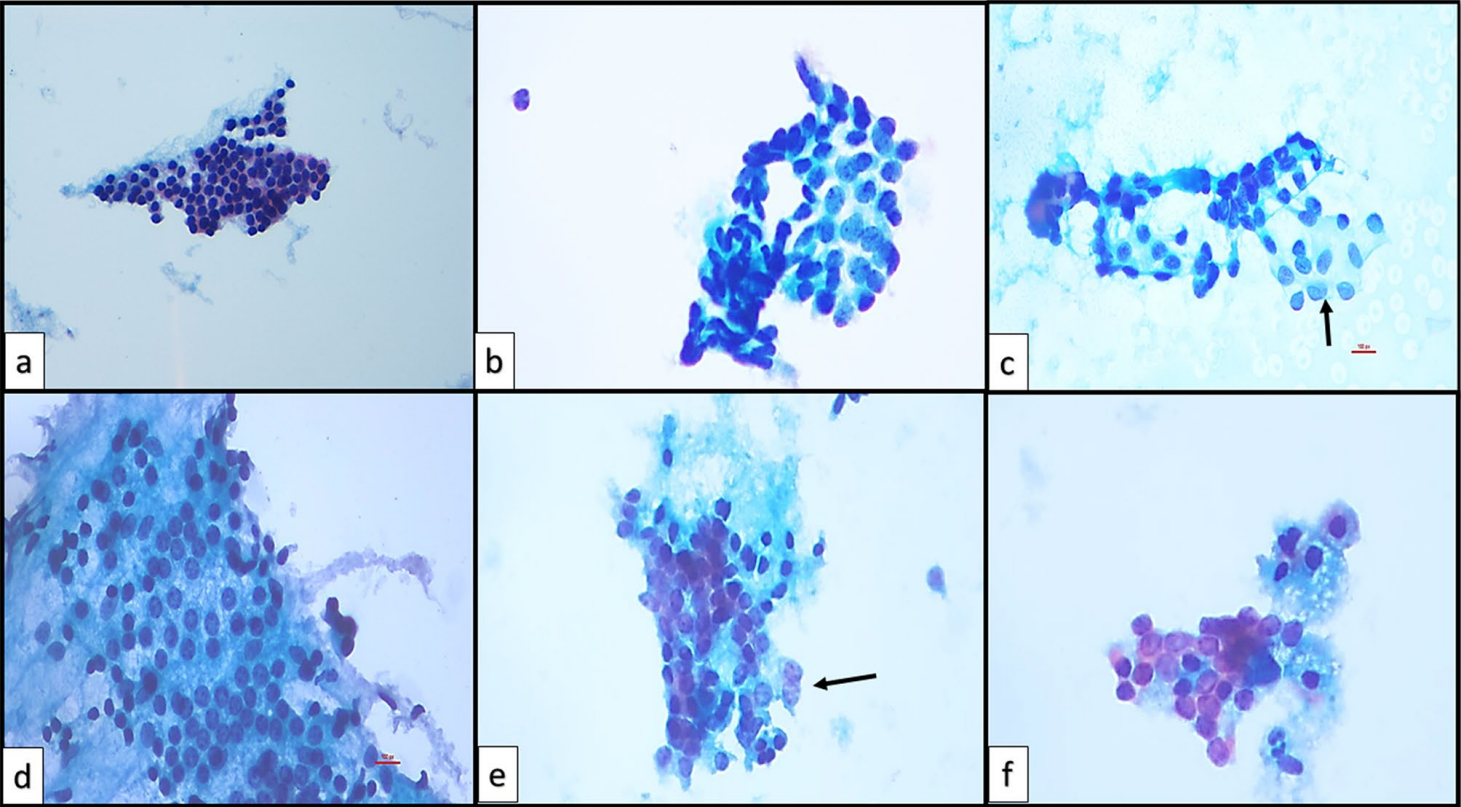
Nuclear scoring. **a** Nuclear score 1 (liquid-based preparation, Papanicolau × 200). **b**, **c**, **e**, **f** Nuclear score 3 (liquid-based preparation, Papanicolau × 400). **d** Nuclear score 2 (liquid-based preparation, Papanicolau × 400)

In order to determine the degree of nuclear atypia, nuclear features were reviewed, and a nuclear scoring was performed, inspired by Maletta et al. [29] and based on the guidelines created by Nikiforov et al. [30] for the histopathological diagnosis of NIFTP. Evaluated nuclear features included the headings as (1) size and shape (nuclear enlargement/overlapping and crowding/molding/elongation), (2) nuclear membrane irregularities (irregular contours/grooves/pseudoinclusions), and (3) chromatin features (chromatin clearing, chromatin margination). Each feature was scored as 0 or 1 depending on its absence/presence, respectively, and total score resulted in a NS ranging from 0 to 3 (Fig. 2). If the NS was 0 or 1, cytological atypia was considered absent, and if the NS was 2 or 3, nuclear atypia was considered present. NS2 was considered mild nuclear atypia, and NS 3 was considered marked nuclear atypia. The presence of a nuclear feature was considered focal when it was present in roughly less than 50% of the sample or when it appeared as a distinct focus from the rest of the sample and as diffuse when it was seen in roughly more than 50% of the sample.

#### Evaluation of Architectural Features

**Fig. 3:**
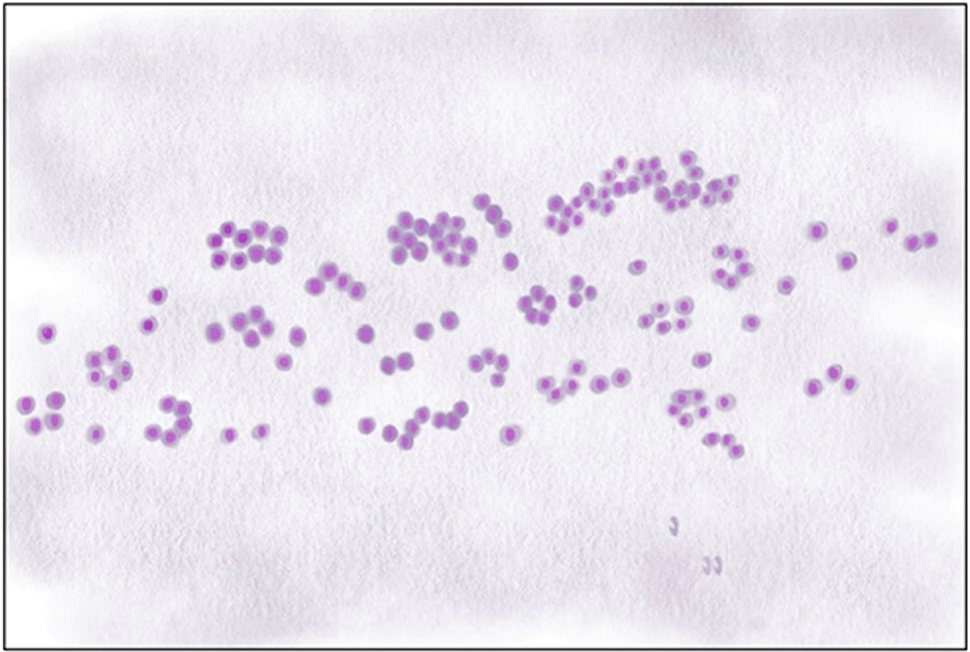
Drawing representing schematic depiction of streaming pattern

Architectural features were evaluated under the headings as in the following: microfollicular pattern, streaming pattern, trabecular pattern, and three-dimensional groups. The presence of one and/or more of these headings at least focally was considered to be “architectural atypia.” The term “streaming pattern” was used for cell populations in a mixed pattern, found in small groups and individual cells in some areas, forming microfollicles and trabeculae, in a continuous relationship with each other, particularly in conventional smears. This property evolved from our observations during the daily practice in evaluating thyroid FNAs. It was called “streaming pattern” because it creates a stream-like appearance in a certain area of the preparation (usually in the midline on the long axis of the preparation) (Fig. 3) (prepared in https://www.sketchbook.com/). The extent of the architectural pattern was considered focal when it involved a single slide or an area of the slide that appeared different from the rest, and otherwise as diffuse (roughly when seen in more than 50% of the aspirate).

### Determination of Subcategories/Subgroups

**Fig. 4:**
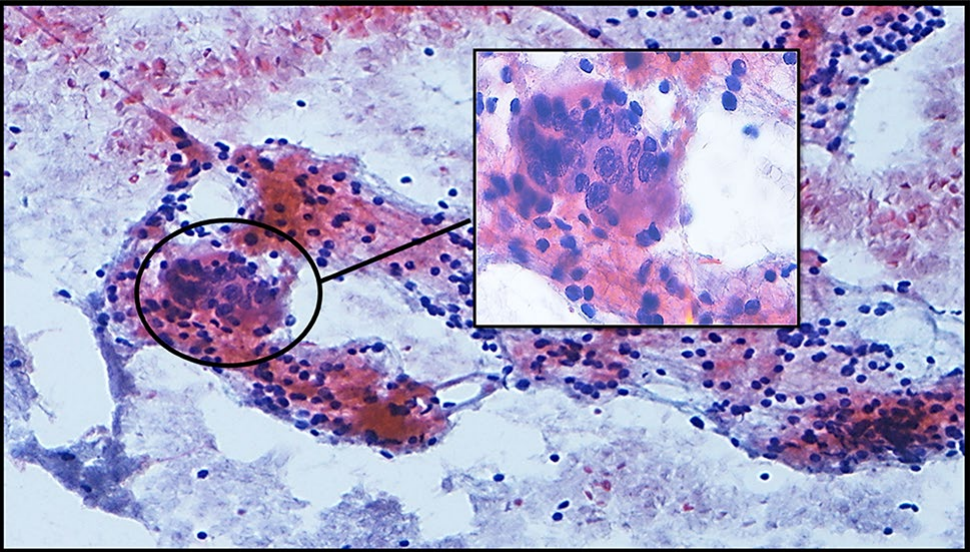
AUS-N1: focal marked nuclear atypia in a background of benign follicular cells (conventional smear, Papanicolau, × 200; inlet, × 400)

**Fig. 5:**
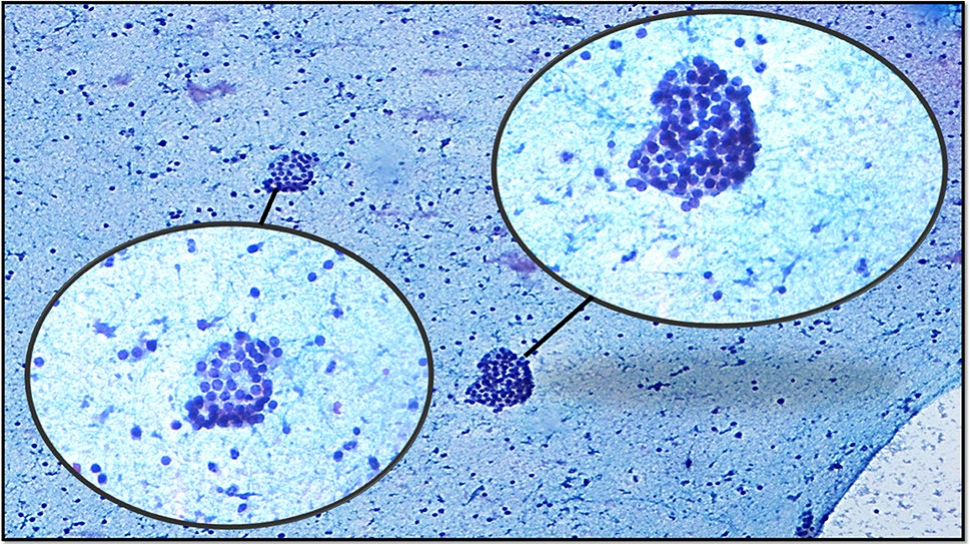
AUS-N1: diffuse nuclear atypia in paucicellular aspirate (liquid-based preparation, Papanicolau, × 200; inlet, × 400)

**Fig. 6:**
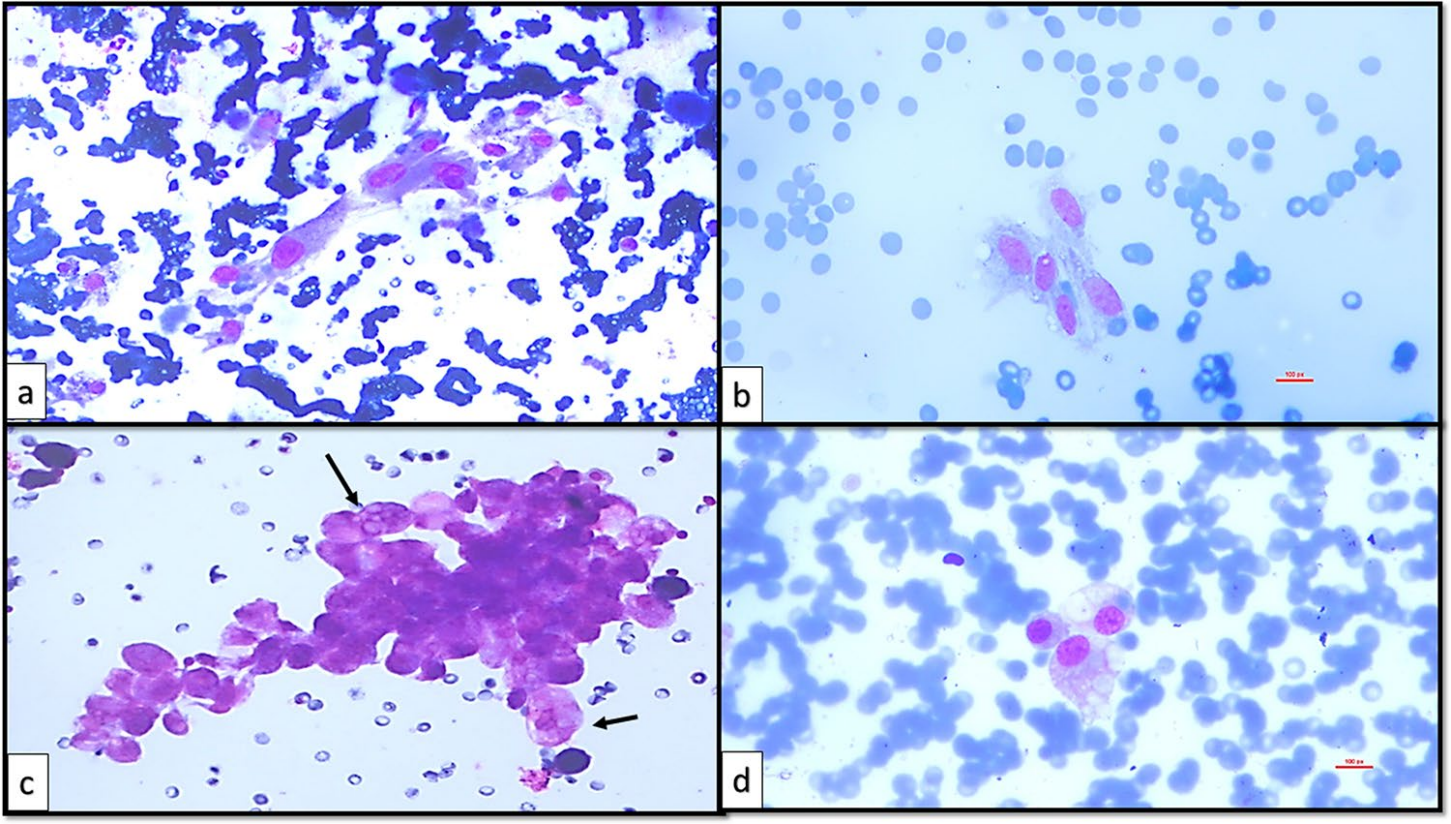
**a**, **b** AUS-N3: atypical cyst-lining cells (conventional smear, MGG, × 400). **c**, **d** AUS-N4: histiocytoid cells (conventional smear, MGG, × 400)

**Fig. 7:**
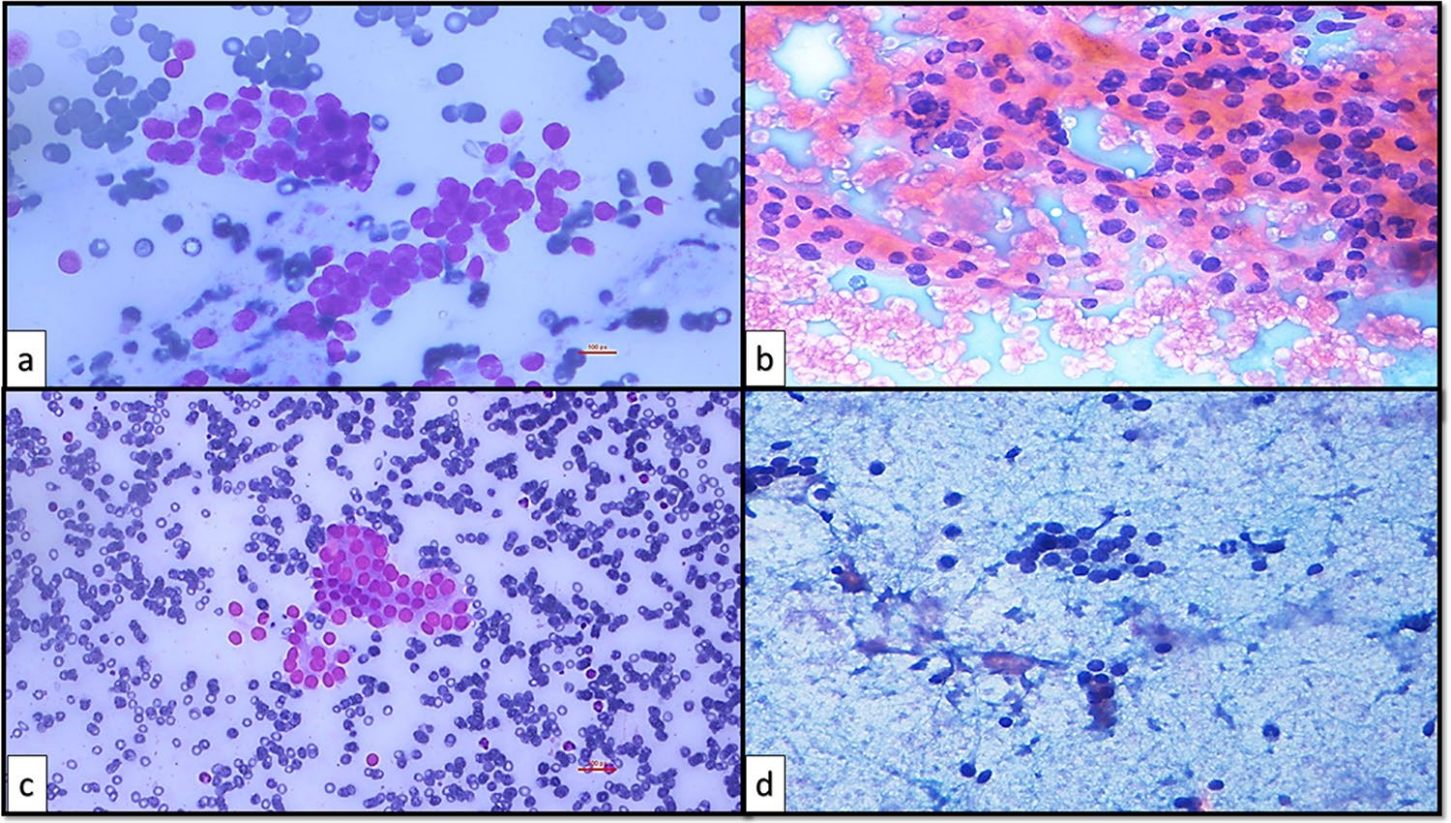
**a**, **b** AUS-N&A: nuclear and architectural atypia (**a** conventional smear, MGG, × 400; **b** conventional smear, Papanicolau, × 400). **c**, **d** AUS-A: architectural atypia (**c** conventional smear, MGG, × 400; **d** conventional smear, Papanicolau, × 400)

**Fig. 8:**
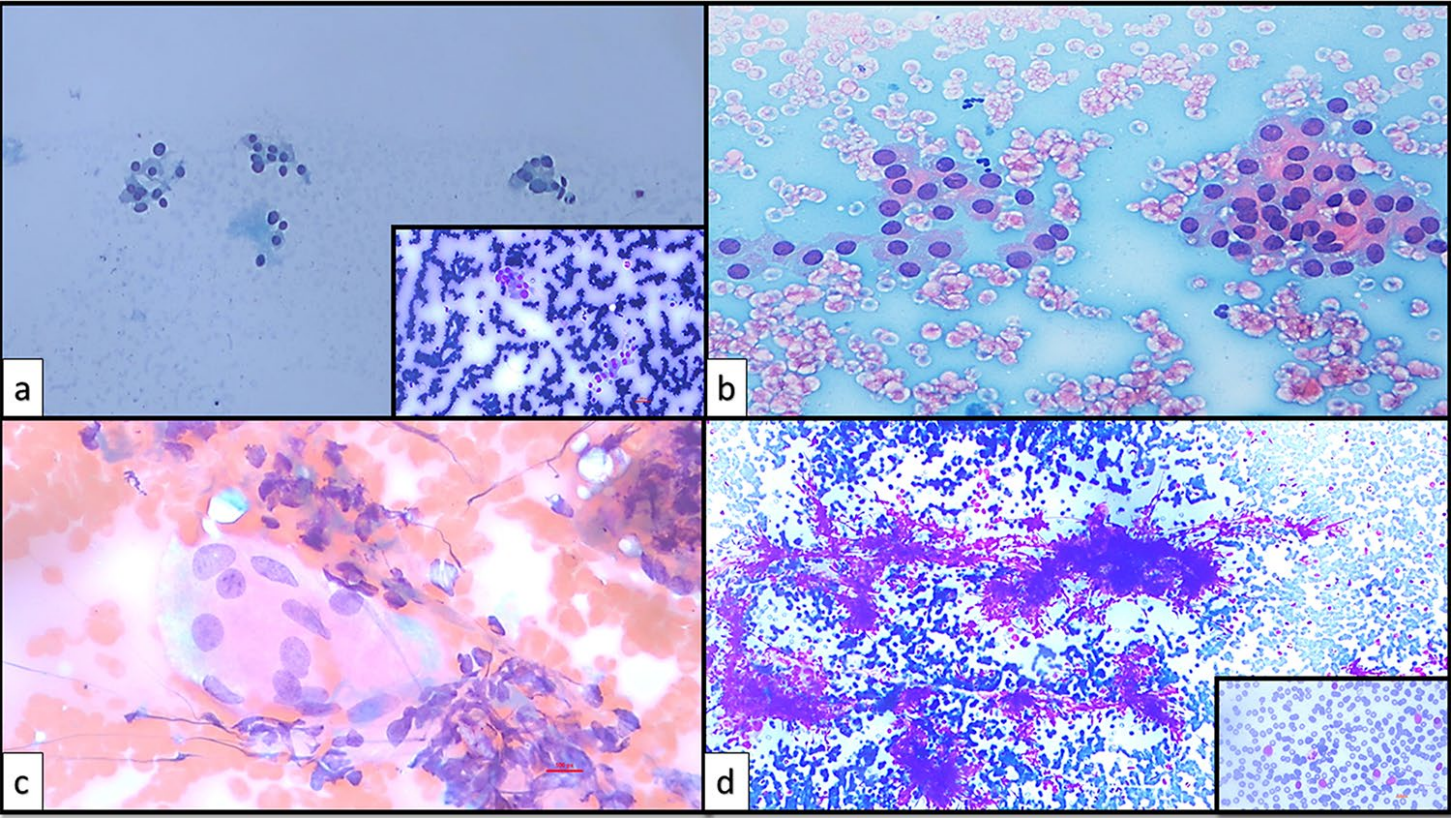
**a** AUS-O1: extensive oncocytic cells with low cellularity (conventional smear, Papanicolau, × 200, inlet; conventional smear, MGG, × 200). **b** AUS-O2: extensive oncocytic cells with intermediate cellularity (conventional smear, Papanicolau, × 200). **c** AUS-NOS1: isolated nuclear enlargement and prominent nucleoli (conventional smear, Papanicolau, × 400). **d** AUS-L: atypical lymphoid cells (conventional smear, MGG, × 40, inlet; conventional smear, MGG, × 400)

The present study was designed in the era of the 2nd edition of TBSRTC [9] and was performed during the transition zone between two editions and finalized after the TBSRTC 3rd edition [6] was reported. The initial subcategories created on the basis of the 2nd edition were revised taking into account the subcategories and criteria expressed in the 3rd edition [6, 9]. Therefore, the subcategorization made in this study covered the criteria reported in both editions of TBSRTC (Table 2). The descriptions expressed about AUS/FLUS in the 2nd edition of TBSRTC [9] and the subcategories with related criteria expressed about AUS in the 3rd edition of TBSRTC [6] were blended, and 6 AUS subcategories and 11 subgroups addressing the possible scenarios that define these subcategories were created as in the following: AUS-nuclear atypia (AUS-N), AUS-architectural atypia (AUS-A), AUS-nuclear and architectural atypia (AUS-N&A), AUS-oncocytic atypia (AUS-A), AUS-not otherwise specified (AUS-NOS), and AUS-lymphoid cells-rule out lymphoma (AUS-L). The subgroups of AUS-N were designed as focal nuclear atypia (AUS-N1) (Fig. 4), diffuse but mild nuclear atypia (AUS-N2) (Fig. 5), atypical cyst-lining cells (AUS-N3) (Fig. 6a, b), and histiocytoid cells (AUS-N4) (Fig. 6c, d). Coexistence of architectural and nuclear atypia was considered subgroup AUS-N&A (Fig. 7a, b). Under the heading of the AUS-A subcategory, two subgroups were determined as in the following: preparations containing diffuse architectural atypia with low cellularity (AUS-A1) (Fig. 7c), preparations containing focal architectural atypia with moderate-marked cellularity (AUS-A2) (Fig. 7d). AUS-O subcategory was divided in two subgroups consisting of AUS-O1 (preparations containing diffuse oncocytic cells with low cellularity) (Fig. 8a) and AUS-O2 (preparations containing diffuse oncocytic cells with intermediate-high cellularity) (Fig. 8b). Three subgroups of AUS-NOS subcategory were created as AUS-NOS1 (preparations with isolated nuclear enlargement and prominence of nucleoli) (Fig. 8c), AUS-NOS2 (preparations including isolated psammomatous calcification), and AUS-NOS3 (preparations with changes that may be due to preparation artifacts and other) (Table 2). AUS-L subgroup included atypical lymphoid cells with suspicion of lymphoma (Fig. 8d).

**Table 2:** Subcategories and subgroups of atypia of undetermined significance

**The present study**		**TBSRTC, 2nd edition **[9]	**TBSRTC, 3rd edition **[6]
**1) Atypia of undetermined significance, nuclear atypia (AUS-N)**	***AUS high-risk group***	1. Cytologic atypia	AUS with nuclear atypia
***AUS-N1 focal nuclear atypia*** • Aspirates showing focal score 3 nuclear atypia without nuclear pseudoinclusions • Aspirates without nuclear pseudoinclusions, low cellularity, and showing diffuse score 3 nuclear atypia		**(a) *Focal cytologic atypia***	***Focal nuclear atypia***
***AUS-N2-common but mild nuclear atypia*** • Aspirates containing score 2 nuclear atypia without nuclear pseudoinclusions		**(b) *Extensive but mild cytologic atypia***	***Extensive but mild nuclear atypia***
***AUS-N3 atypical cyst-lining cells*** • Aspirates containing atypical-appearing cyst-lining cells due to nuclear clefts, prominent nucleoli, elongated nuclei and extended cytoplasm, and/or rare intranuclear pseudoinclusions in a generally benign specimen		**(c) *Atypical cyst-lining cells***	***Atypical cyst-lining cells***
***AUS-N4 histiocytoid cells*** • Aspirates including histiocytoid cells containing rounder nuclei, a higher nuclear-to-cytoplasmic ratio, and “harder” (glassier) cytoplasm, without the hemosiderin or microvacuolization of histiocytes, though larger, discrete vacuoles		(**d) *“Histiocytoid” cells***	***“Histiocytoid” cells***
**2) Atypia of undetermined significance, nuclear and architectural atypia (AUS-N&A)** • Coexistence of architectural and nuclear atypia		**2. Cytologic and architectural atypia**	**Nuclear and architectural atypia**
			**AUS-other**
**3) Atypia of undetermined significance, architectural atypia (AUS-A)**	***AUS low-risk group***	**3. Architectural atypia**	**Architectural atypia**
***AUS-A1 diffuse architectural atypia, low cellularity*** • Aspirates with low cellularity, containing a small amount of colloids, a nuclear score of 0 or 1, and widespread architectural atypia		**(a)** A scantly cellular specimen with rare clusters of follicular cells, almost entirely in microfollicles or crowded three-dimensional groups and with scant colloid	**1.** A scantly cellular specimen with rare clusters of follicular cells, almost entirely in microfollicles or crowded three-dimensional groups and with scant colloid
***AUS-A2 focal architectural atypia, moderate to marked cellularity*** • Aspirates with moderate or marked cellularity, nuclear score of 0 or 1, and focal architectural atypia		**(b)** Focally prominent microfollicles with minimal nuclear atypia	**2.** A moderately to markedly cellular specimen exhibits architectural atypia as described above in most follicular cells (50–70% of follicular cells) but without a marked predominance (at least 70% of follicular cells) that would warrant a FN diagnosis
			**3.** Focally prominent microfollicles without nuclear atypia. A more prominent than usual population of microfollicles may be seen in a moderately or markedly cellular sample or in the clinical setting of MNG, but the overall proportion of microfollicles is not sufficient for a diagnosis of FN
**4) Atypia of undetermined significance, oncocytic atypia (AUS-O)**		**4. Hürthle cell aspirates**	**Oncocytic/oncocyte atypia**
***AUS-O1 Diffuse oncocytic cells, low cellularity*** • Aspirates consisting of diffuse oncocytic cells with low cellularity and small amounts of colloids		**(a)** A sparsely cellular aspirate comprised exclusively (or almost exclusively) of Hürthle cells with minimal colloid	**1**. A sparsely cellular aspirate comprised exclusively or almost exclusively of oncocytic (previously termed Hürthle) cells with minimal colloid
***AUS-O2 diffuse oncocytic cells, moderate to prominent cellularity*** • Aspirates with moderate to prominent cellularity, consisting of diffuse oncocytic cells, suggesting the possibility of benign lesion such as lymphocytic thyroiditis, but containing insufficient lymphocytes for a definitive diagnosis		**(b)** A moderately or markedly cellular sample composed exclusively (or almost exclusively) of Hürthle cells, yet the clinical setting suggests a benign Hürthle cell nodule, such as in lymphocytic (Hashimoto) thyroiditis or a multinodular goiter (MNG)	**2**. A moderately or markedly cellular sample composed exclusively or almost exclusively of oncocytic cells (at least 70% of all follicular cells), in which the clinical setting suggests a benign oncocytic cell nodule, such as in lymphocytic (Hashimoto) thyroiditis or a multinodular goiter (MNG)
**5) Atypia of undetermined significance, not otherwise specified (AUS-NOS)**		**5. Atypia, not otherwise specified (NOS)**	**Atypia, not otherwise specified (NOS)**
***AUS-NOS1*** • Aspirates showing nuclear enlargement and prominence of nucleoli in some of the follicle cells		**(a)** A minor population of follicular cells shows nuclear enlargement, often accompanied by prominent nucleoli	**1**. A minor population of follicular cells shows nuclear enlargement, often accompanied by prominent nucleoli
***AUS-NOS2*** • Presence of psammomatous calcifications in smears with a nuclear score of 0 or 1		**(b)** Psammomatous calcifications in the absence of nuclear features of papillary carcinoma	**2.** Psammomatous calcifications in the absence of follicular cells with nuclear features of papillary carcinoma
***AUS-NOS3*** • Aspirates containing changes that may be due to preparation artifact (Including cases in which the observed cytological changes cannot be clearly determined as artificial or real atypia)		**(c)** Rare instances of atypia warranting an AUS/FLUS designation not explicitly described elsewhere in this chapter	**3.** Rare instances of atypia warranting an AUS designation not explicitly described elsewhere in this chapter
**6) Atypia of undetermined significance, atypical lymphoid cells (AUS-L)** • Aspirates containing atypical lymphoid cells		**6. Atypical lymphoid cells, rule out lymphoma** There is an atypical lymphoid infiltrate (for which a repeat aspirate for flow cytometry is desirable), but the degree of atypia is insufficient for the general category “suspicious for malignancy”	**Atypical lymphoid cells, rule out lymphoma** There is an atypical lymphoid infiltrate for which a repeat aspirate for flow cytometry is desirable; however, the degree of atypia is insufficient for the general category of “suspicious for malignancy.” Besides lymphoma, other tumors such as thymic lesions may be in the differential diagnosis

*AUS-N* atypia of undetermined significance, nuclear atypia; *AUS-A* atypia of undetermined significance, architectural atypia; *AUS-N&A* atypia of undetermined significance, nuclear and architectural atypia; *AUS-O* atypia of undetermined significance, oncocytic atypia; *AUS-NOS* atypia of undetermined significance, not otherwise specified; *AUS-L* atypia of undetermined significance, atypical lymphoid cells

### Determination of ROM

The ROM was calculated as a percentages obtained by dividing the total number of histopathologically confirmed malignant cases by the AUS cases with surgical follow-ups. The ROM was calculated separately for each subcategory/subgroup/NS/nuclear feature/architectural feature.

### Statistical Analysis

Results were shown as mean ± Std. Deviation or numbers and percentages. The chi-square test (Pearson, continuity correction, or Fisher) was used in comparisons of categorical variables. Multivariate logistic regression analysis was used to examine the effect of nuclear features on histological malignancy, and odds ratio and 95% confidence intervals were calculated. ROC analysis was used to examine the discriminative power of nuclear features on malignancy, and AUC, cutoff, sensitivity, and specificity values were calculated. *p* < 0.05 value was accepted as the limit value of statistical significance. SPSS 20.0 statistical package program was used to analyze the data (IBM SPSS Statistics for Windows, version 20.0. Armonk, NY, IBM Corp.).

## Results

The clinicopathological features of the study group are presented in Table 1. The mean age of the study population was 50.1 ± 11.9 years. The ratio of men to women was found to be 1.00:3.56. Nodule sizes ranged from 0.5 to 7.5 cm, with the average size being 1.5 cm. The rate of AUS was 17.6%. Overall risk of malignancy for AUS was 30.7%. According to histopathological diagnosis after resection, 13 (5%) of the cases were diagnosed as NND, 138 (53.1%) were diagnosed as BN, 29 (11.2%) were diagnosed as LRN, and 80 (30.7%) were in the malignant category (Fig. 1).

### Comparisons of Nuclear Features and NS with Histopathological Diagnosis and ROM

#### Comparisons of Nuclear Features with Histopathological Diagnosis and ROM

**Fig. 9:**
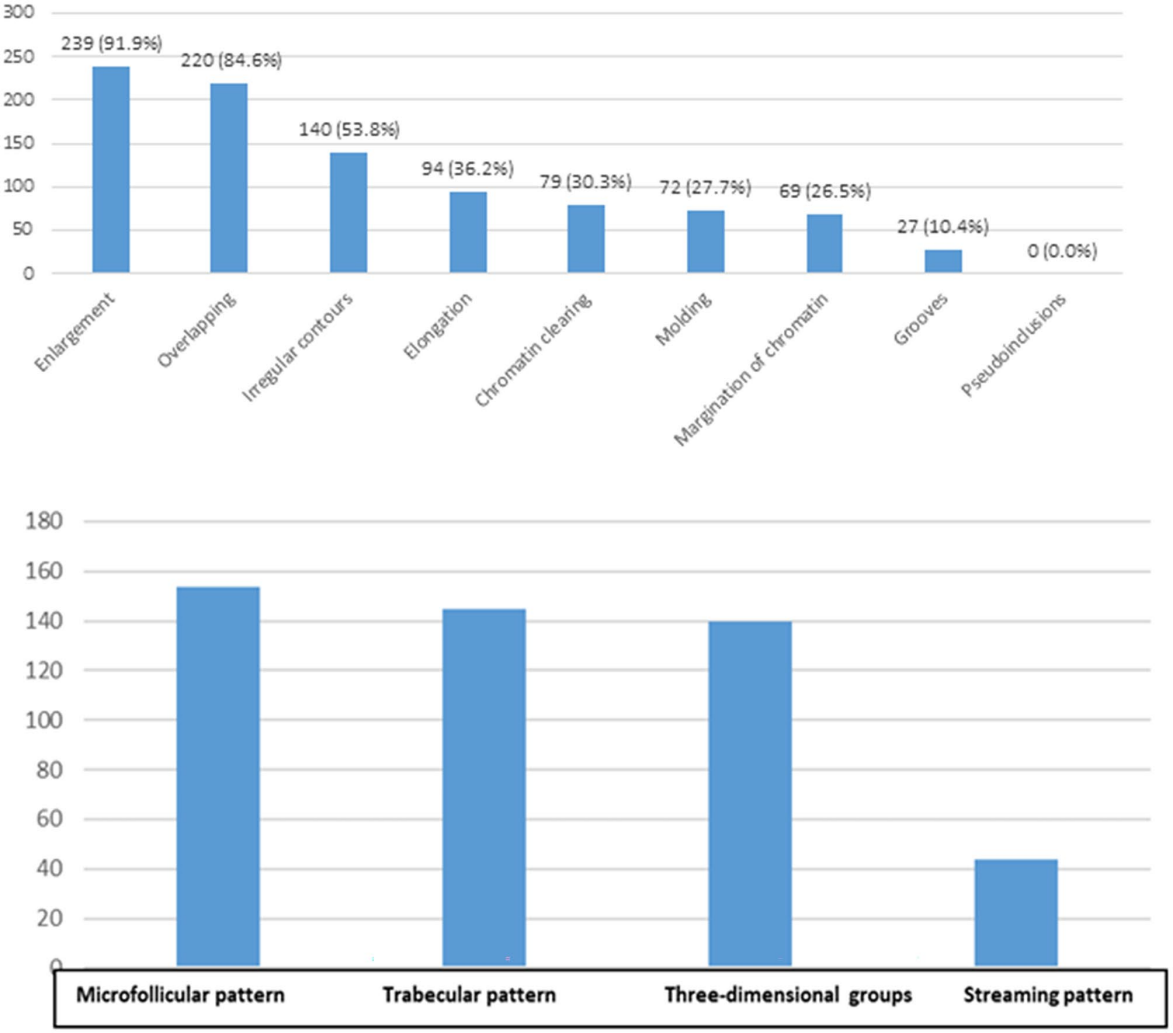
Frequency of nuclear and architectural features in the study group

The most frequently observed nuclear feature in FNAs was nuclear enlargement which was seen in 239 (91.9%) of the cases. Descending order for frequency of other nuclear features was as follows: nuclear crowding, nuclear membrane irregularities, nuclear elongation, chromatin clearing, molding, chromatin margination, and nuclear grooves. No intranuclear pseudoinclusion was observed (Fig. 9). Comparisons of nuclear features with histopathological diagnosis are presented in Table 3. Nuclear overlapping, chromatin clearing, and margination of nuclear chromatin were significantly more common in LRNs and MNs (*p* < 0.001), while nuclear molding, nuclear contour irregularity, and nuclear grooves were significantly more frequent in MNs (*p* < 0.001). The ROM was significantly higher in the FNAs with nuclear overlapping (35.5%, *p* < 0.001), with nuclear molding (56.9%, *p* < 0.001), with nuclear contour irregularity (42.1%, *p* < 0.001), with nuclear grooves (74.1%, *p* < 0.001), with chromatin clearing (49.4%, *p* < 0.001), and with chromatin margination (57.7%, *p* = 0.004) than the FNAs without these features. The ROM was higher in patients with nuclear enlargement with a close to the significance (32.6%, *p* = 0.051) (Table 3).

**Table 3:** Relationship between nuclear features with histopathological diagnosis and risk of malignancy

**Nuclear features**		**Nonneoplastic disease *n* (%)**	**Benign neoplasm *n* (%)**	**Low-risk neoplasm *n* (%)**	**Malignant neoplasm *n* (%)**	**Total *n* (%)**	***p* value**	**Risk of malignancy (%)**	***p* value**
**Nuclear enlargement**	**Present**	12 (92.3)	122 (51.0)	27 (11.3)	78 (32.6)	239 (100.0)	0.093*	32.6	0.051**
	**Absent**	1 (4.8)	16 (76.2)	2 (9.5)	2 (9.5)	21 (100.0)		9.5	
**Nuclear elongation**	**Present**	5 (5.3)	45 (47.9)	10 (10.6)	34 (36.2)	94 (100.0)	0.530***	36.2	0.156***
	**Absent**	8 (4.8)	93 (56.0)	19 (11.4)	46 (27.8)	166 (100.0)		27.7	
**Nuclear overlapping**	**Present**	10 (4.5)	104 (47.3)	28 (12.7)	78 (35.5)	220 (100.0)	< 0.001*	35.5	< 0.001**
	**Absent**	3 (7.5)	34 (85.0)	1 (2.5)	2 (5.0)	40 (100.0)		5.0	
**Nuclear molding**	**Present**	2 (2.8)	21 (29.2)	8 (11.1)	41 (56.9)	72 (100.0)	< 0.001***	56.9	< 0.001***
	**Absent**	11 (5.9)	117 (62.2)	21 (11.2)	39 (20.7)	188 (100.0)		20.7	
**Nuclear contour irregularity**	**Present**	6 (4.3)	61 (43.6)	14 (10.0)	59 (42.1)	140 (100.0)	< 0.001***	42.1	< 0.001**
	**Absent**	7 (5.8)	77 (64.2)	15 (12.5)	21 (17.5)	120 (100.0)		17.5	
**Nuclear grooves**	**Present**	0 (0.0)	5 (18.5)	2 (7.4)	20 (74.1)	27 (10.0)	< 0.001*	74.1	< 0.001**
	**Absent**	13 (5.6)	133 (57.1)	27 (11.6)	60 (25.7)	233 (100.0)		25.8	
**Chromatin clearing**	**Present**	2 (2.5)	26 (32.9)	12 (15.2)	39 (49.4)	79 (100.0)	< 0.001***	49.4	< 0.001***
	**Absent**	11 (6.1)	112 (61.9)	17 (9.4)	41 (22.6)	181 (100.0)		22.7	
**Margination of chromatin**	**Present**	1 (3.8)	2 (7.7)	8 (30.8)	15 (57.7)	26 (100.0)	< 0.001*	57.7	0.004**
	**Absent**	12 (5.1)	136 (58.1)	21 (9.0)	65 (27.8)	234 (100.0)		34.0	

^*^Fisher’s exact test, ^**^continuity correction, ^***^Pearson chi-square

#### Impact of Nuclear Features on ROM

**Fig. 10:**
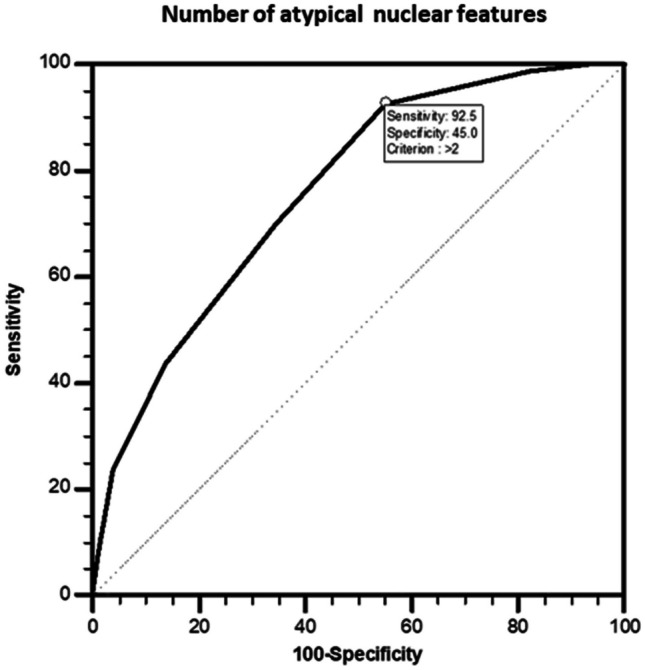
ROC analysis for likelihood of malignancy according to the number of nuclear features

The effect of nuclear features on histopathological malignancy was evaluated by logistic regression analysis. Multivariate analysis revealed that nuclear grooves [6.3 (2.5–16.0), *p* < 0.001], nuclear overlapping/crowding [6.2 (1.4–27.1, *p* = 0.016], nuclear molding [3.9 (2.1–7.1), *p* < 0.001], irregular nuclear contours [2.8 (1.5–5.1), *p* = 0.001], chromatin clearing [2.6 (1.4–4.8), *p* = 0.002], and margination of chromatin [2.5 (1.1–6.1), *p* = 0.035] were significant independent predictors for histological malignancy (Table 4). And also, ROC analysis revealed that presence of > 2 nuclear features [AUC = 0.760 (standard error = 0.0299); *p* < 0.0001] may have likelihood of histological malignancy (Table 5, Fig. 10).

**Table 4:** Impact of nuclear features on histological malignancy by multivariate logistic regression analysis

			**Malignant disease**		***p***	**Odds ratio (95% confidence interval)**
			**Absent** (*n* = 180) *n* (%)	**Present** (*n* = 80) *n* (%)		
**Nuclear size and shape**	**Nuclear enlargement**	**Absent**	19 (90.5)	2 (9.5)	0.440	1.8 (0.4–8.7)
		**Present**	161 (67.4)	78 (32.6)		
	**Nuclear elongation**	**Absent**	120 (72.3)	46 (27.7)	0.846	1.1 (0.6–1.9)
		**Present**	60 (63.8)	34 (36.2)		
	**Nuclear overlapping/crowding**	**Absent**	38 (95.0)	2 (5.0)	**0.016**	6.2 (1.4–27.1)
		**Present**	142 (64.5)	78 (35.5)		
	**Nuclear molding**	**Absent**	149 (79.3)	39 (20.7)	** < 0.001**	3.9 (2.1–7.1)
		**Present**	31 (43.1)	41 (56.9)		
**Nuclear membrane irregularities**	**Nuclear contour irregularity**	**Absent**	99 (82.5)	21 (17.5)	**0.001**	2.8 (1.5–5.1)
		**Present**	81 (57.9)	59 (42.1)		
	**Nuclear grooves**	**Absent**	173 (74.2)	60 (25.8)	** < 0.001**	6.3 (2.5–16.0)
		**Present**	7 (25.9)	20 (74.1)		
**Chromatin characteristics**	**Chromatin clearing**	**Absent**	145 (75.9)	46 (24.1)	**0.002**	2.6 (1.4–4.8)
		**Present**	35 (50.7)	34 (49.3)		
	**Margination of chromatin**	**Absent**	169 (72.2)	65 (27.8)	**0.035**	2.5 (1.1–6.1)
		**Present**	11 (42.3)	15 (57.7)		

Nagelkerke *R*^2^ values are 0.021 for “nuclear size and shape,” 0.184 for “nuclear membrane irregularities,” 0.099 for “chromatin characteristics”

**Table 5:** Discriminative power of nuclear features on malignancy

Nuclear features	Sensitivity (%)	95% confidence interval	Specificity (%)	95% confidence interval	Likelihood ratio +	Likelihood ratio −
** > 0**	100.00	95.5–100.0	6.67	3.5–11.4	1.07	0.00
** > 1**	98.75	93.2–100.0	17.78	12.5–24.2	1.20	0.070
> 2	**92.50**	**84.4**–**97.2**	**45.00**	**37.6–52.6**	**1.68**	**0.17**
** > 3**	70.00	58.7–79.7	65.56	58.1–72.5	2.03	0.46
** > 4**	43.75	32.7–55.3	86.11	80.2–90.8	3.15	0.65
** > 5**	23.75	14.9–34.6	96.11	92.2–98.4	6.11	0.79
** > 6**	8.75	3.6–17.2	98.89	96.0–99.9	7.88	0.92
** > 7**	1.25	0.03–6.8	100.00	98.0–100.0	-	0.99

AUC = 0.760 (standard error = 0.0299); *p* < 0.0001

#### Comparisons of NS with Histopathological Diagnosis and ROM

Comparisons of NS according to histopathological diagnosis revealed that NS1 (*p* = 0.001), either extensive NS1 (*p* = 0.031) or focal NS1 (*p* = 0.005), was significantly more common in BNs than other diagnostic categories. Also, NS1 was more common in NND (*p* = 0.001). Focal NS2 was significantly more frequent in BNs (*p* = 0.034). NS3, either focal or extensive, was significantly detected at higher rates in MNs (*p* < 0.001). The ROM was significantly higher in the FNAs with NS3 (64.2%, *p* < 0.001), in FNAs with focal NS3 (61.1%, *p* < 0.001), and in FNAs with extensive NS3 (70.6%, *p* = 0.001). However, significant lower ROM was detected in FNAs with NS0 (0.0%, *p* = 0.020), in FNAs with NS1 (16.7%, *p* = 0.001), in FNAs with focal NS1 (12.2%, *p* = 0.009), and in FNAs with focal NS2 (17.3%, *p* = 0.029) (Table 6).

**Table 6:** Relationship between nuclear score with histopathological diagnosis and risk of malignancy

**Nuclear score**		**Nonneoplastic disease *n* (%)**	**Benign neoplasm *n* (%)**	**Low-risk neoplasm *n* (%)**	**Malignant neoplasm *n* (%)**	**Total *n* (%)**	***p* value**	**Risk of malignancy (%)**	***p* value**
**Nuclear score 0**	**Present**	0 (0.0)	9 (81.8)	2 (18.2)	0 (0.0)	11 (100.0)	0.061*	0.0	0.020*
	**Absent**	13 (5.2)	129 (51.8)	27 (10.9)	80 (32.1)	249 (100.0)		32.1	
**Nuclear score 1**	**Present**	7 (8.3)	56 (66.7)	7 (8.3)	14 (16.7)	84 (100.0)	0.001**	16.7	0.001***
	**Absent**	6 (3.4)	82 (46.6)	22 (12.5)	66 (37.5)	176 (100.0)		37.5	
**Nuclear score 1—focal**	**Present**	1 (2.4)	32 (78.0)	3 (7.3)	5 (12.2)	41 (100.0)	0.005*	12.2	0.009***
	**Absent**	12 (5.5)	106 (48.4)	26 (11.9)	75 (34.2)	219 (100.0)		34.2	
**Nuclear score 1—extensive**	**Present**	6 (14.0)	24 (55.8)	4 (9.3)	9 (20.9)	43 (100.0)	0.031*	20.9	0.177***
	**Absent**	7 (3.3)	114 (52.5)	25 (11.5)	71 (32.7)	217 (100.0)		32.7	
**Nuclear score 2**	**Present**	4 (3.6)	63 (56.3)	13 (11.6)	32 (28.6)	112 (100.0)	0.677**	28.6	0.595***
	**Absent**	9 (6.1)	75 (50.7)	16 (10.8)	48 (32.4)	148 (100.0)		32.4	
**Nuclear score 2—focal**	**Present**	2 (3.8)	37 (71.2)	4 (7.7)	9 (17.3)	52 (100.0)	0.034**	17.3	0.029***
	**Absent**	11 (5.3)	101 (48.6)	25 (12.0)	71 (34.1)	208 (100.0)		34.1	
**Nuclear score 2—extensive**	**Present**	2 (3.3)	26 (43.3)	9 (15.0)	23 (38.3)	60 (100.0)	0.230**	38.3	0.198***
	**Absent**	11 (5.5)	112 (56.0)	20 (10.0)	57 (28.5)	200 (100.0)		28.5	
**Nuclear score 3**	**Present**	2 (3.8)	10 (18.9)	7 (13.2)	34 (64.2)	53 (100.0)	< 0.001**	64.2	< 0.001***
	**Absent**	11 (5.3)	128 (61.8)	22 (10.6)	46 (22.3)	207(100.0)		22.2	
**Nuclear score 3—focal**	**Present**	2 (5.6)	9 (25.0)	3 (8.3)	22 (61.1)	36 (100.0)	< 0.001*	61.1	< 0.001***
	**Absent**	11 (4.9)	129 (57.6)	26 (11.6)	58 (25.9)	224(100.0)		25.9	
**Nuclear score 3—extensive**	**Present**	0 (0.0)	1 (5.9)	4 (23.5)	12 (70.6)	17 (100.0)	< 0.001*	70.6	0.001***
	**Absent**	13 (5.3)	137 (56.4)	25 (10.3)	68 (28.0)	243(100.0)		28.0	

^*^Fisher’s exact test, ^**^Pearson chi-square, ^***^Continuity correction

#### Comparisons of Architectural Features with Histopathological Diagnosis and ROM

The most common architectural feature was the microfollicular pattern which was detected in 154 (59.2%) of the cases. Trabecular pattern was seen in 145 (55.8%) of the cases, three-dimensional groups were seen in 140 (53.8%) of the cases, and streaming pattern was present in 44 (16.9%) of the cases. Microfollicular pattern (*p* = 0.026) and trabecular pattern (*p* = 0.048) were significantly more common in BNs and in LRNs than other diagnostic categories. However, three-dimensional groups was significantly more frequent in LRNs and MNs (*p* = 0.018). Also, the ROM was significantly lower in FNAs with microfollicular pattern (26.0%, *p* = 0.043) (Table 7).

**Table 7:** Relationship between architectural features with histopathological diagnosis and risk of malignancy

**Architectural features**		**Nonneoplastic disease *n* (%)**	**Benign neoplasm *n* (%)**	**Low-risk neoplasm *n* (%)**	**Malignant neoplasm *n* (%)**	**Total *n* (%)**	***p* value**	**Risk of malignancy (%)**	***p* value**
**Microfollicular pattern**	**Present**	5 (3.2)	87 (56.5)	22 (14.3)	40 (26.0)	154 (100.0)	0.026*	26.0	0.043*
	**Absent**	8 (7.5)	51 (48.1)	7 (6.6)	40 (37.7)	106 (100.0)		37.7	
**Trabecular pattern**	**Present**	4 (2.8)	80 (55.2)	21 (14.5)	40 (27.6)	145 (100.0)	0.048*	27.6	0.212*
	**Absent**	9 (7.8)	58 (50.4)	8 (7.0)	40 (34.8)	115 (100.0)		34.8	
**Three-dimensional groups**	**Present**	3 (2.1)	68 (48.6)	19 (13.6)	50 (35.7)	140 (100.0)	0.018*	35.7	0.062*
	**Absent**	10 (8.3)	70 (58.4)	10 (8.3)	30 (25.0)	120 (100.0)		25.0	
**Streaming pattern**	**Present**	1 (2.3)	28 (63.6)	5 (11.4)	10 (22.7)	44 (100.0)	0.459**	22.7	0.276***
	**Absent**	12 (6.1)	110 (56.1)	24 (12.3)	50 (25.5)	196 (100.0)		32.4	

^*^Pearson chi-square, ^**^Fisher’s exact test, ^***^Continuity correction

#### Comparisons of AUS Subcategories and Subgroups with Histopathological Diagnosis and ROM

Comparisons of AUS subcategories and subgroups with histopathological diagnosis are presented in Table 8. According to the comparisons, AUS-N subcategory (48.2%, *p* = 0.014) was significantly more common in MNs, while AUS-N1 subgroup was also significantly more common in MNs (65.2%) (*p* < 0.001). FNAs with AUS-A subcategory (71.9%, *p* = 0.002) and AUS-A2 (77.8%, *p* = 0.015) subgroup properties were significantly more frequent in BNs. Subcategory AUS-N&A was significantly more common in LRNs (16.0%) and MNs (38.3%) (*p* = 0.006). FNAs with subcategory AUS-O and subgroup AUS-O2 properties significantly presented as NND (*p* < 0.001). Subcategory AUS-NOS was significantly related with histological diagnosis as NND and BN (*p* = 0.016). The ROM was significantly higher in FNAs with properties of AUS-N (48.2%, *p* = 0.001), AUS-N1 (65.2%, *p* < 0.001), and AUS-N&A (38.3%, *p* = 0.048) than FNAs without these features. On the other hand, the ROM was significantly lower in FNAs with features of subcategories AUS-A (17.2%, *p* = 0.011), AUS-0 (5.6%, *p* = 0.033), and AUS-NOS (11.5%, *p* = 0.044). In the AUS-N&A subcategory, comparisons were made between NS2 and NS3 in terms of ROM. According to these comparisons, ROM in the AUS-N&A subcategory with NS3 features was significantly higher than in the AUS-N&A subcategory with NS2 findings (*p* = 0.006) (Table 9).

**Table 8:** Relationship between AUS subcategories with histopathological diagnosis and risk of malignancy

**AUS subcategories**		**Nonneoplastic disease *n* (%)**	**Benign neoplasm *n* (%)**	**Low-risk neoplasm *n* (%)**	**Malignant neoplasm *n* (%)**	**Total *n* (%)**	***p* value**	**Risk of malignancy (%)**	***p* value**
**AUS-N**	**Present**	2 (3.6)	21 (37.5)	6 (10.7)	27 (48.2)	56 (100.0)	0.014*	48.2	0.001*
	**Absent**	11 (5.4)	117 (57.4)	23 (11.3)	53 (25.9)	204 (100.0)		26.0	
**AUS-N1**	**Present**	0 (0.0)	4 (17.4)	4 (17.4)	15 (65.2)	23 (100.0)	< 0.001**	65.2	< 0.001***
	**Absent**	13 (5.5)	134 (56.5)	25 (10.6)	65 (27.4)	237 (100.0)		27.4	
**AUS-N2**	**Present**	2 (8.3)	13 (54.2)	2 (8.3)	7 (29.2)	24 (100.0)	0.821**	29.2	1.000 ***
	**Absent**	11 (4.7)	125 (53.0)	27 (11.4)	73 (30.9)	236 (100.0)		30.9	
**AUS-N3**	**Present**	0 (0.0)	2 (66.7)	0 (0.0)	1 (33.3)	3 (100.0)	1.000**	33.3	1.000**
	**Absent**	13 (5.1)	136 (52.9)	29 (11.3)	79 (30.7)	257 (100.0)		30.7	
**AUS-N4**	**Present**	0 (0.0)	2 (33.3)	0 (0.0)	4 (66.7)	6 (100.0)	0.407**	66.7	0.074**
	**Absent**	13 (5.1)	136 (53.5)	29 (11.4)	76 (29.9)	254 (100.0)		29.9	
**AUS-A**	**Present**	0 (0.0)	46 (71.9)	7 (10.9)	11 (17.2)	64 (100.0)	0.002*	17.2	0.011***
	**Absent**	13 (6.6)	92 (47.0)	22 (11.2)	69 (35.2)	196 (100.0)		35.2	
**AUS-A1**	**Present**	0 (0.0)	18 (64.3)	5 (17.9)	5 (17.9)	28 (100.0)	0.170**	17.9	0.177***
	**Absent**	13 (5.6)	120 (51.7)	24 (10.3)	75 (32.3)	232 (100.0)		32.3	
**AUS-A2**	**Present**	0 (0.0)	28 (77.8)	2 (5.6)	6 (16.7)	36 (100.0)	0.015**	16.7	0.075***
	**Absent**	13 (5.8)	110 (49.1)	27 (12.1)	74 (33.3)	224 (100.0)		33.0	
**AUS-N&A**	**Present**	1 (1.1)	42 (44.7)	15 (16.0)	36 (38.3)	94 (100.0)	0.006*	38.3	0.048*
	**Absent**	12 (7.2)	96 (57.8)	14 (8.5)	44 (26.5)	166 (100.0)		26.5	
**AUS-O**	**Present**	7 (38.9)	10 (55.6)	0 (0.0)	1 (5.6)	18 (100.0)	< 0.001**	5.6	0.033***
	**Absent**	6 (2.5)	128 (52.9)	29 (12.0)	79 (32.6)	242 (100.0)		32.6	
**AUS-O1**	**Present**	1 (11.1)	7 (77.8)	0 (0.0)	1 (11.1)	9 (100.0)	0.240**	11.1	0.282**
	**Absent**	12 (4.8)	131 (52.2)	29 (11.6)	79 (31.4)	251 (100.0)		31.5	
**AUS-O2**	**Present**	6 (66.7)	3 (33.3)	0 (0.0)	0 (0.0)	9 (100.0)	< 0.001**	0.0	0.061**
	**Absent**	7 (2.8)	135 (53.8)	29 (11.6)	80 (31.9)	251 (100.0)		31.9	
**AUS-NOS**	**Present**	3 (11.5)	19 (73.1)	1 (3.8)	3 (11.5)	26 (100.0)	0.016**	11.5	0.044***
	**Absent**	10 (4.2)	119 (50.9)	28 (12.0)	77 (32.9)	234 (100.0)		32.9	
**AUS-NOS 1**	**Present**	1 (143)	5 (71.4)	1 (14.3)	0 (0.0)	7 (100.0)	0.128**	0.0	0.104**
	**Absent**	12 (4.7)	133 (52.6)	28 (11.1)	80 (31.6)	253 (100.0)		31.6	
**AUS-NOS 2**	**Present**	0 (0.0)	0 (0.0)	0 (0.0)	0 (0.0)	0 (100.0)	**_**	0.0	**-**
	**Absent**	13 (5.0)	138 (53.0)	29 (11.2)	80 (30.8)	260 (100.0)		30.7	
**AUS-NOS 3**	**Present**	2 (10.5)	14 (73.7)	0 (0.0)	3 (15.8)	19 (100.0)	0.064**	15.8	0.226***
	**Absent**	11 (4.6)	124 (51.4)	29 (12.0)	77 (32.0)	241 (100.0)		32.0	
**AUS-L**	**Present**	0 (0.0)	0 (0.0)	0 (0.0)	2 (100.0)	2 (100.0)	**_**	100.0	**-**
	**Absent**	13 (5.0)	138 (53.5)	29 (11.2)	78 (30.3)	258 (100.0)		30.2	

^*^Pearson chi-square, ^**^Fisher’s exact test,^***^Continuity correction

*AUS* atypia of undetermined significance, *AUS-N* atypia of undetermined significance-nuclear atypia, *AUS-A* atypia of undetermined significance-architectural atypia, *AUS-N&A* atypia of undetermined significance-nuclear atypia and architectural atypia, *AUS-O* atypia of undetermined significance-oncocytic atypia, *AUS-NOS* atypia of undetermined significance-not otherwise specified, *AUS-L* atypia of undetermined significance-lymphoid cells

**Table 9:** Relationship between risk of malignancy and nuclear scores in subgroup AUS-N&A

**Nuclear score (focal/extensive)**	**Risk of malignancy % (***n***)**	***p* value**	**Nuclear score**	**Risk of malignancy % (***n***)**	***p* value**
Focal nuclear score 2	15.6 (5)	0.052*	**NS2**	28.1 (18)	0.006*
Extensive nuclear score 2	40.6 (13)				
Focal nuclear score 3	50.0 (9)	0.260**	**NS3**	60.0 (18)	
Extensive nuclear score 3	75.0 (9)				

^*^Continuity correction, ^**^Fisher’s exact test

*AUS-N&A* atypia of undetermined significance-nuclear atypia and architectural atypia

#### Pairwise Comparisons Between Subcategories of AUS According to the ROM and Identification of New Risk Groups

Pairwise comparisons between subcategories of AUS according to the ROM are presented in Table 10. No statistically significant difference was observed in terms of malignancy risk between the subcategories AUS-N and AUS-N&A (*p* = 0.308). The ROM for subcategory AUS-N was significantly higher than subcategories including AUS-A (*p* = 0.001), AUS-NOS (*p* = 0.003), and AUS-O (*p* = 0.003). There were similar results in comparisons for the AUS-N&A subcategory with other subcategories. AUS-N&A subcategory revealed higher ROM than subcategories such as AUS-A (*p* = 0.008), AUS-NOS (*p* = 0.019), and AUS-O (*p* = 0.015).

**Table 10:** Pairwise comparison of malignancy risks in AUS subcategories

	**Benign disease *n* (%)**	**Malignant disease *n* (%)**	**Total *n* (%)**	***p* value**
AUS-N	29 (51.8)	27 (48.2)	56 (100.0)	0.308*
AUS-N&A	58 (61.7)	36 (38.3)	94 (100.0)	
AUS-N	29 (51.8)	27 (48.2)	56 (100.0)	0.001*
AUS-A	53 (82.8)	11 (17.2)	64 (100.0)	
AUS-N	29 (51.8)	27 (48.2)	56 (100.0)	0.003*
AUS-NOS	23 (88.5)	3 (11.5)	26 (100.0)	
AUS-N	29 (51.8)	27 (48.2)	56 (100.0)	0.003*
AUS-O	17 (94.4)	1 (5.6)	18 (100.0)	
AUS-N&A	58 (61.7)	36 (38.3)	94 (100.0)	0.008*
AUS-A	53 (82.8)	11 (17.2)	64 (100.0)	
AUS-N&A	58 (61.7)	36 (38.3)	94 (100.0)	0.019*
AUS-NOS	23 (88.5)	3 (11.5)	26 (100.0)	
AUS-N&A	58 (61.7)	36 (38.3)	94 (100.0)	0.015*
AUS-O	17 (94.4)	1 (5.6)	18 (100.0)	
AUS-A	53 (82.8)	11 (17.2)	64 (100.0)	0.749**
AUS-NOS	23 (88.5)	3 (11.5)	26 (100.0)	
AUS-A	53 (82.8)	11 (17.2)	64 (100.0)	0.286**
AUS-O	17 (94.4)	1 (5.6)	18 (100.0)	
AUS-NOS	23 (88.5)	3 (11.5)	26 (100.0)	0.634**
AUS-O	17 (94.4)	1 (5.6)	18 (100.0)	

^*^Continuity correction, ^**^Fisher’s exact test

*AUS* atypia of undetermined significance, *AUS-N* atypia of undetermined significance-nuclear atypia, *AUS-A* atypia of undetermined significance-architectural atypia, *AUS-N&A* atypia of undetermined significance-nuclear and architectural atypia, *AUS-O* atypia of undetermined significance-oncocytic atypia, *AUS-NOS* atypia of undetermined significance-not otherwise specified, *AUS-L* atypia of undetermined significance-lymphoid cells

Since the malignancy risks of AUS-N and AUS-N&A subcategories are significantly higher than the malignancy risks of other subcategories (AUS-A, AUS-O, AUS-NOS), AUS-N and AUS-N&A subcategories are considered a distinct group, and the other subcategories are considered a separate group. The ROM for two groups was compared. These comparisons revealed that the ROM was significantly higher in the group including AUS-N and AUS-N&A subcategories than the group including other subcategories (42.0% vs 13.9%) (*p* < 0.001). So, the group consisting of subcategories with higher risk of malignancy was named as “high-risk group” and the group including other subcategories with lower ROM was named as “low-risk group” (Table 11). Finally, ROM values were compared between AUS and “suspicious for malignancy” (SFM) categories (ROM value for SFM category was obtained from the archive records without reevaluation of aspirates). ROM of SFM was calculated in 103 nodules of 103 patients with surgical follow-ups. ROM for SFM (95.1%) was significantly higher than ROM values for AUS (overall), AUS-nuclear subcategory, AUS-other subcategory, and AUS-NS3 subgroup (Table 12).

**Table 11:** Comparison of malignancy risks in high-risk AUS group and low-risk AUS group

	**Benign disease *n* (%)**	**Malignant disease *n* (%)**	**Total *n* (%)**	***p* value**
High-risk AUS group (AUS-N and AUS-N&A)	87 (58.0)	63 (42.0)	150 (100.0)	< 0.001*
Low-risk AUS group (AUS-A, AUS-NOS, AUS-O, AUS-L)	93 (84.5)	17 (15.5)	110 (100.0)	

^*^Pearson chi-square

*AUS* atypia of undetermined significance, *AUS-N* atypia of undetermined significance-nuclear atypia, *AUS-A* atypia of undetermined significance-architectural atypia, *AUS-N&A* atypia of undetermined significance-nuclear and architectural atypia, *AUS-O* atypia of undetermined significance-oncocytic atypia, *AUS-NOS* atypia of undetermined significance-not otherwise specified, *AUS-L* atypia of undetermined significance-lymphoid cells

**Table 12:** Pairwise comparison of malignancy risks between AUS vs SFM, AUS-nuclear vs SFM, AUS-other vs SFM, and AUS-NS3 vs SFM

	**Benign disease *n* (%)**	**Malignant disease *n* (%)**	**Total *n* (%)**	***p* value**
AUS	180 (69.3)	80 (30.7)	260 (100.0)	< 0.001*
SFM	5 (4.9)	98 (95.1)	103 (100.0)	
AUS-nuclear	87 (58.0)	63 (42.0)	150 (100.0)	< 0.001*
SFM	5 (4.9)	98 (95.1)	103 (100.0)	
AUS-other	93 (84.5)	17 (15.5)	110 (100.0)	< 0.001*
SFM	5 (4.9)	98 (95.1)	103 (100.0)	
AUS-NS3	19 (35.8)	34 (64.2)	53 (100.0)	< 0.001*
SFM	5 (4.9)	98 (95.1)	103 (100.0)	

^*^Fisher’s exact test

*AUS* atypia of undetermined significance, *SFM* suspicious for malignancy, *NS* nuclear score

## Discussion

After the introduction of the ultrasound into the clinical evaluation of thyroid diseases in the late 1960s, the frequency of detected thyroid nodules has been increased due to the widespread use of ultrasonography [1, 2]. Ultrasound-guided FNA cytology serves to provide the most appropriate clinical management of thyroid nodules. TBSRTC including six categories with suggestions of appropriate clinical management for each category provides a standardized reporting format for thyroid FNAs which has been updated with significant developments during the last two decades following its first edition [6]. However, indeterminate categories continue to exist including probably the most problematic category “Atypia of Undetermined Significance (Bethesda III)” in the 3rd edition of the reporting system [6]. In this context, the present study aimed to investigate the impact of AUS subcategories and subgroups designed according to the criteria reported in the 2nd [9] and in the 3rd edition [6] of TBSRTC on ROM via a three-staged study plan. In the first stage, nuclear and architectural features were evaluated, and these features were compared with the histopathological diagnostic groups determined according to the diagnostic categories and tumor types reported in the 5th edition (2022 Beta version) of the WHO Classification of Endocrine and Neuroendocrine Tumours [28], and comparisons of ROM were performed between all of the features. In the second stage, the current study tried to develop a nuclear scoring schema inspired by the study of Nikiforov et al. [30]. Finally, in the third stage, subcategories and subgroups were created based on NS, other cellular features, and architectural features, and these subcategories and subgroups were compared in terms of histopathological diagnosis and ROM. So, the most remarkable results of the present study can be summarized as in the following: (i) The umbrella term as AUS-nuclear subcategory, particularly subgroups including focal marked nuclear atypia (focal NS 3) and nuclear atypia with architectural atypia, constitutes a high-risk group in terms of malignancy; (ii) nuclear features including nuclear grooves, nuclear overlapping, molding, nuclear contour irregularity, chromatin clearing, and margination are independent significant predictors of malignancy; (iii) three-tiered nuclear scoring scheme may provide a more objective and reproducible assessment method in the evaluation of thyroid FNAs as in the assessment of histological nuclear features.

Although 3rd edition of TBSRTC [6] continues to suggest an upper limit as 10% for AUS in all thyroid FNAs, the reported rates range between 1 [10] and 20% [23] (Table 13). The rate of AUS was 17.6% in the current study (This value reflects the general data in our department. It does not include the evaluations made in the present study.), and this value was higher than the proposed rate of AUS in TBSRTC 3rd edition [6]. Probable cause of the higher rate of AUS may be the subjective application of the defined objective criteria in the evaluation of thyroid FNAs. Also, in each edition, the rate of ROM was updated according to the accumulated data and the effects of changing terminologies [6]. However, the reported rates of ROM in the literature have been often higher than the recommended values during the age of TBSRTC (Table 13). The ROM for categories was reported as mean and expected ranges in the recent edition of TBSRTC and ROM for AUS was reported as 22% (13–30) [6]. The current gold standard calculation of ROM is obtained by dividing the total number of histopathologically confirmed malignant cases by the AUS cases with surgical follow-ups; however, the calculation method is controversial since approximately half of the thyroid nodules in the AUS category do not have surgical follow-ups. Most of the data reported about the rates of ROM in the literature are the results of the studies performed before the era of NIFTP and ranges between 17.0 [15] and 83.1% [16]. Liu et al. [8] excluded NIFTP from the malignant diagnostic category and informed ROM as 54.3% in their study. In the present study, histological diagnostic categories were created according to the 5th edition (Beta version) of the WHO Classification of Endocrine and Neuroendocrine Tumours [28], and NIFTP was categorized as a LRN and was not considered a malignant tumor in the calculation of ROM. The risk of malignancy was 30.7% in the present study and was slightly higher than the upper value of the expected range in the 3rd edition of TBSRTC [6] but lower than the rate reported by Liu et al. [8]. The higher rate of ROM in the present study may be due to the selection of high-risk nodules for surgery by evaluating patients diagnosed as AUS with clinical and radiological findings in a multidisciplinary manner. So, wide range between the reported results for the rates of AUS and ROM, which mostly represent real-life data, may be related to various factors, including inadequate clinicopathological communication, insufficient practice of objective criteria for evaluation of FNAs, the tendency of observers to stay in the safe zone, and the lack of experience of researchers.

**Table 13:** Review of the previous studies investigating the effect of AUS subcategories on risk of malignancy

**Study**	**Year**	**Country**	**Number of patients**	**Number of nodules/FNAs**	**Histological diagnosis**	**AUS rate (%)**		**Histological malignant disease (%)**	**Histological benign disease (%)**
**Renshaw **[17]	2010	USA	548	-	204	14	Overall	25.0	75.0
							Atypical cells; rule out papillary carcinoma	38.0	62.0
							Atypical cells; rule out Hurthle cell neoplasm	7.0	93.0
							Atypical cells; rule out follicular neoplasm	22.0	78.0
							Atypia; not otherwise specified	27.0	73.0
**Olson et al. **[18]	2011	USA	133	-	133	9.8	Overall	32.0	68.0
							AUS-N; focal nuclear atypia	48.0	52.0
							AUS-F; focal microfollicular features	27.0	73.0
							AUS-O; not otherwise specified	12.0	88.0
**VanderLaan et al. **[19]	2011	USA	331	331 nodules	199	10.9	Overall	48.2	51.8
							AUS-architectural atypia	24.0	76.0
							AUS-cytologic atypia	50.0	50.0
							AUS-cytologic and architectural atypia	46.0	54.0
							AUS-unspecified	64.0	36.0
**Luu et al. **[44]	2011	USA	222	222 FNAs	127	3.1	Overall	26.0	74.0
							AFLUS-cannot exclude PTC	45.8	54.2
							AFLUS-other patterns	13.9	86.1
**Horne et al. **[15]	2012	USA	171	-	58	2.8	Overall	17.0	83.0
							INa; low cellularity, predominant microfollicular pattern, no or minimal colloid	7.0	93.0
							INb; nuclear atypia	56.0	44.0
**Önder et al. **[21]	2014	Turkey	399	421 FNAs	103	6.7	Overall	18.9	81.1
							AUS-PTC pattern	28.0	72.0
							AUS-microfollicle pattern	6.9	93.1
							AUS-Hurthle cell pattern	0.0	100.0
							AUS-atypical cell pattern	22.2	77.8
**Wu et al. **[23]	2014	USA	138	-	138	20.0	Overall	33.0	67.0
							AUS-PTC; cannot exclude papillary thyroid carcinoma (high-risk group)	54.0	46.0
							AUS-FN; cannot exclude follicular neoplasm (high-risk group)	34.0	66.0
							AUS-HCN; cannot exclude Hurthle cell neoplasm (low-risk group)	18.0	82.0
							AUS-NOS; not otherwise specified (low-risk group)	19.0	81.0
**Chen et al. **[20]	2014	USA	72	82 FNAs	39	18.0	Overall	18.0	82.0
							3- or 4-tiered scoring model for general features, other cells, and atypia	-	-
**Hyeon et al. **[45]	2014	Korea	551	-	231	8.6	Overall	68.0	32.0
							AUS-nuclear atypia	78.8	21.2
							FLUS-architectural atypia	13.2	86.8
**Mathur et al. **[39]	2014	USA	806	-	255	17.0	Overall	39.0	61.0
							AUS-N; focal nuclear atypia	54.0	46.0
							AUS-F; focal microfollicular features	39.0	61.0
							AUS-HC; predominance of Hurthle cells	19.0	81.0
							AUS-O; others	26.0	74.0
**Park et al. **[22]	2014	Korea	331	-	95	9.6	Overall	81.1	76.7
							AUS-NA; focal nuclear atypia	97.1	2.9
							AUS-MF; predominantly microfollicular features	22.2	77.8
							AUS-HC; focal Hurthle cell features	20.0	80.0
							AUS-O; others	61.5	38.5
**Shrestha et al. **[46]	2016	USA	221	-	101	10.0	Overall	29.0	71.0
							AUS-cellular atypia	35.0	65.0
							AUS-architectural atypia	10.0	90.0
							AUS-Hurthle cells predominant	33.0	67.0
							AUS-others	0.0	100.0
**Kim et al. **[26]	2017	Korea	94	-	33	6.5	Overall	43.6	56.4
							AUS-N; nuclear atypia	100.0 (18/18)	0.0
							AUS-A; architectural atypia	45.5 (7/11)	55.5
							AUS-O; predominant oncocytic changes	100.0 (3/3)	0.0
							AUS-N/A; both nuclear and architectural atypia	100.0 ( 1/1)	0.0
**Gan et al. **[25]	2017	Singapore	309	-	137	6.4 (unpublished)	Overall	27.0	73.0
							AUS/FLUS; nuclear atypia	36.8	63.2
							AUS/FLUS; architectural atypia	14.7	85.3
							AUS/FLUS; lymphoid atypia	100.0 ( 5/5)	0.0
**Kim et al. **[47]	2017	Korea	660	-	68	12.3	Overall	40.3	57.7
							AUS-cellular atypia	48.2	51.8
							AUS-architectural atypia	14.2	85.8
**Roy et al. **[37]	2019	India	117	-	47	13.0	Overall	78.7	21.3
							AUS; architectural atypia	73.3	26.7
							AUS; cytological atypia	78.3	21.7
							AUS; architectural and cytological atypia	71.4	28.6
							AUS; with Hürthle cells	100.0 (1/1)	0.0
							AUS; not otherwise specified	100.0 (1/1)	0.0
**Zhao et al. **[16]	2021	China	1053	-	195	8.2	Overall	83.1	16.9
							Cytologic atypia 1 (AUS-N1)	92.6	7.4
							Cytologic atypia 2 (AUS-N2)	66.7	33.3
							Architectural atypia (AUS-A)	0.0	100.0
							Cytologic and architectural atypia (AUS-N&A)	84.0	16.0
							Hürthle cell aspirates (AUS-H)	61.5	38.5
							Atypia, not otherwise specified (AUS-NOS)	36.4	63.6
							Atypical lymphoid cells, (AUS-L)	66.7	33.3
**Liu et al. **[8]	2022	China	184	188	188	11.2	Overall	54.3	45.7
							Nuclear atypia	65.9	34.1
							Architectural atypia	50.7	49.3
**Babajani et al. **[38]	2023	Iran	70	-	41	8.8	Overall	61.0	39.0
							Architectural atypia	0.0	100.0
							Cytologic atypia	57.1	42.9
							Cytologic and architectural atypia	59.1	40.9
							Hürthle cell AUS/FLUS	21.4	78.6
							Atypia which was not specified	40.0	60.0
**Guerreiro et al. **[48]	2023	Portugal	-	79	79	16.0	Overall	44.3	55.7
							AUS-nuclear atypia	50.0	50.0
							AUS-other	43.2	56.8
**The present study,** **Bagıs et al.**	2023	Turkey	240	260	260	17.6	Overall	30.7	69.3
							AUS-nuclear atypia (4 subgroups)	48.2	51.8
							AUS-architectural atypia (2 subgroups)	17.2	82.8
							AUS-nuclear and architectural atypia	38.3	61.7
							AUS-oncocytic atypia (2 subgroups)	5.6	94.4
							AUS-NOS (3 subgroups))	11.5	88.5
							AUS-lymphoid atypia	100.0	0.0
							High-risk AUS group	42.0	58.0
							Low-risk AUS group	13.9	86.1

The present study investigated the impact of nuclear and architectural features on risk of malignancy in FNAs with AUS. All of patients in the study group had surgical follow-ups, and the FNA localization and the target lesion in the surgical specimen overlapped. The first FNAs of each nodule were included in the study in order to prevent previous FNA-related regenerative changes from affecting the evaluation. Microfollicular pattern and trabecular pattern were seen significantly more frequently in FNAs of BNs and LRNs, while three-dimensional groups were significantly more common in FNAs of MNs in surgical specimens. And also, three-dimensional groups show the highest ROM value with close to significance. Gularia et al. [31] and Kaymaz et al. [32] showed that cases containing a three-dimensional group had higher ROM values. So, a more skeptical investigation of FNAs with three-dimensional groups for an additional evidence of malignancy may be argued. Additionally, definition of streaming pattern which was detected in 44 cases in our study is reported for the first time in the literature. Although, a partially similar pattern is mentioned in the TBSRTC 3rd edition (architectural atypia, criteria 3) [6], it is not thought to fully correspond to the pattern described here. When we examined the cases with streaming patterns, it was determined that initial FNAs of BNs more frequently exhibited this pattern without statistically significance. However, considering that all of the cases in our study were in the AUS category, our findings may not clearly reflect the nature of the streaming pattern, and investigation of this pattern in a study group with a larger number of cases including all of the TBSRTC categories may reveal the impact of this pattern on thyroid cytology.

Comparisons of nuclear features with ROM values revealed that the risk of malignancy in FNAs with nuclear grooves, chromatin margination, nuclear molding, nuclear contour irregularity, and nuclear overlapping (in an order with descending ROM values) was significantly higher than in FNAs without these features. Also, nuclear grooves, nuclear overlapping, molding, nuclear contour irregularity, chromatin clearing, and chromatin margination (in an order with descending Odds ratios) were significant independent predictors of histological malignancy in multivariate analysis. However, nuclear overlapping, chromatin clearing, and chromatin margination were also more common in the group consisting of histological LRNs. There was no relationship between nuclear elongation and nuclear enlargement with histological malignancy. Kato et al. [33] reported that the presence of nuclear grooves may indicate histological malignancy in indeterminate thyroid cytology. Additionally, the authors stated that the presence of four or more atypical nuclear features or coexistence of nuclear grooves and inclusions may be associated with malignancy. Kaymaz et al. stated that membrane irregularities such as pseudoinclusion, nuclear contour irregularity, and nuclear grooves were associated with malignancy, and also, nuclear elongation and overlapping were predictive for malignancy [32]. FNAs with nuclear grooves revealed the highest risk of malignancy, and in multivariate analysis, the presence of nuclear grooves resulted as an independent significant predictor for malignancy with the highest value of odds ratio as the authors have reported. On the other hand, the presence of more than two of six atypical nuclear features was found to be a significant likelihood of malignancy in the ROC analysis performed in this study. The previous results reported in terms of the relationship between malignancy with nuclear contour irregularity, nuclear overlapping, and nuclear grooves were similar with the results of the present study regarding these parameters. However, according to the definition in TBSRTC [6, 9], pseudoinclusion should by definition not be present in the evaluation of nuclear atypia for AUS except atypical cyst-lining cells. In the current study, there was no FNA revealing intranuclear pseudoinclusion in the study group. As a result, the presence of nuclear features investigated in the current study may be valuable criteria for marked nuclear atypia. Also, the presence of nuclear grooves or the presence of more than two of the mentioned atypical nuclear features may represent likelihood of malignancy.

The difficulty in using subcategories, as well as the difficulty in using the diagnostic category of AUS, is evidenced by the wide range of ROM reported in the previous studies (Table 13). These differences may probably result from changes in the identification of variables and the entities over time. However, the striking point is the presence of interobserver variability in the evaluation of cytological atypia despite the reported widespread definitions and the criteria. Although the features called cytological or nuclear atypia (such as nuclear enlargement, chromatin clearing, and nuclear membrane irregularity) are the same among cytopathologists, there is significant subjectivity in the evaluation and standardization is quite weak. In the recent history of endocrine pathology, definition of nuclear scoring for nuclear features has made a significant contribution to the evaluation of encapsulated follicular patterned thyroid tumors with papillary-like nuclear features [30]. So, the current study tried to develop a nuclear scoring schema inspired by the study of Nikiforov et al. [30] in order to reduce this subjectivity in the evaluation of nuclear features. Thus, nuclear features were evaluated under the headings of (1) size and shape, (2) nuclear membrane irregularities, and (3) chromatin features resulting in a NS ranging from 0 to 3. Among the NS groups, the group with the highest ROM with a rate of 64.2% (*n* = 34) is the NS3 group. Compared to the overall ROM in the present study (30.7%), the malignancy risk of focal/diffuse NS3 was above this rate and was significantly higher than the other NSs. In this context, focal or diffuse NS3 was found to be more noteworthy in cytological evaluation. The NS2 was significantly more common in FNAs of BNs. Guleria et al. [31] and Kaymaz et al. [32] used similar nuclear scoring models as in the current study and showed that the NS2-3 group was more frequently associated with malignancy. However, in these studies, ROM of NSs were not presented separately, and their focal/diffuse status was not evaluated. Altınboğa et al. [34] examined aspirates with AUS according to the NS, but the scoring system they used was different from the scoring system used in the present study and was designed according to the percentages of nuclear features, and scores were given between 0 and 10 points for nuclear features. For this reason, the relationship between NS and ROM could not be compared with the results reported by the authors. However, there are also studies examining the cytological features of NIFTP using similar scoring system [29, 35, 36]. According to the data of these studies, which evaluated cytological features in TBSRTC indeterminate categories, it can be inferred that cases with NS2-3 have a higher ROM compared to cases with NS0-1. Evaluation of nuclear features by a scoring schema may provide a more objective and safer evaluation method in the categorization and subcategorization of thyroid FNAs, as it can transform the detected findings into numerical data.

The TBSRTC categorization and reported recommendations for each category standardizes the reporting of thyroid cytology and the clinical management of thyroid nodules. However, the criteria that will be reflected in the microscope objective for each category are defined in detail; the interpretation of what is reflected from the microscope eyepieces varies among pathologists. The fact that most of the studies published since the first edition reported values above the recommended rates of AUS and ROM by TBSRTC is a reflection of these variabilities. In the current study, which was carried out during the transition zone between the TBSRTC 2nd edition [9] and TBSRTC 3rd edition [6], 6 subcategories and 11 subgroups [AUS-N (AUS-N1-4), AUS-A (A1-2), AUS-N&A, AUS-O (O1-2), AUS-NOS (NOS1-3), and AUS-L] were created based on the definitions and criteria reported for each category in the TBSRTC 2nd edition [9], updated according to according to the reported terms in the 3rd edition of TBSRTC [6] and powered by the nuclear scoring schema, in order to contribute to a more objective evaluation. So, the definitions of subcategories and subgroups in the present study enclosed the successor and predecessor criteria of TBSRTC (Table 2) [6, 9].

Accumulated data from published studies reporting ROM for AUS following the 1st edition [7] resulted in subcategories as AUS-nuclear and AUS-other in the 3rd edition [6]. The subcategories reported in some of the studies on this subject in the literature and the malignancy rates evaluated in surgical resection materials for each category are summarized in Table 13. In these studies, there are different terminologies that may cover the AUS-nuclear subcategory and subcategories with different numbers and definitions that may correspond to the AUS-other subcategory. In subcategories that may include the AUS-nuclear subcategory, ROM varies between 28 [21] and 100% [26]. While some of these mentioned studies evaluated architectural atypia separately, some examined it within other subcategories. Additionally, reported ROM values for architectural atypia vary between 6.9 [21] and 73.3% [37].

In this study, the AUS-nuclear subcategory covers the cytological atypia criteria in the TBSRTC 2nd edition [9]; it includes definitions other than the nuclear and architectural atypia criteria defined under the AUS-nuclear atypia subcategory in the TBSRTC 3rd edition [6]. ROM of the AUS-N subcategory was significantly higher than the other subcategories (AUS-L subcategory was ignored due to the low number of cases). Therefore, if the results of the current study and the data accumulated from previous studies are combined, the superiority of the AUS-N subcategory over other subcategories in predicting histological malignancy is clear, and the subcategorization reported in the TBSRTC 3rd edition [6] may be foreseeable to benefit in the clinical management of thyroid nodules diagnosed with AUS. In this case, the question may emerge: Does every nuclear atypia express this prediction correctly? In order to clarify this question, the AUS-N subcategory was examined separately in four subgroups in this study. AUS-N1 subgroup had higher ROM than other AUS-N subgroups and all other subcategories and subgroups in the study. In other words, in FNAs with focal NS3 features, ROM is high, and this value was found to be 65.2% in this study. This value is close to the ranges [74 (67–83%)] reported in the TBSRTC 3rd edition [6] for “Suspicious for malignancy, Bethesda Category V.” Perhaps, it may be questioned that upgrading of FNAs with NS 3/AUS-N1 subgroup as SFM rather than the AUS category and the use of the AUS diagnostic category may be reduced. Therefore, ROM values were compared between AUS and SFM categories in the current study (ROM value for SFM category was obtained from the archive records without reevaluation of aspirates). ROM of SFM (95.1%) was significantly higher than ROM values for AUS (overall), AUS-nuclear subcategory, AUS-other subcategory, and AUS-NS3 subgroup. The higher ROM of SFM in our department was approximately within the recommended rates for Bethesda category VI, malignant [97% (97–100)] [6]. Lack of the minimum quantitative threshold for a diagnosis of malignancy in FNAs and also protective effect of the term as “suspicious” may be the causes of the higher rate of ROM in SFM. The quality (presence/absence of intranuclear pseudoinclusions) and quantity (extent of intranuclear pseudoinclusions for malignancy) of the nuclear features and the perception of these features on the pathologists may cause the same features to be evaluated in different TBSRTC categories by different pathologists. Although presence of a few intranuclear pseudoinclusions and/or extensive nuclear grooves in follicular cells and widespread of other atypical nuclear features (approximately > 70% of aspirates) was regarded as SFM rather than AUS in the present study, presented ROM for SFM obtained from the archive records may reflect the interobserver variability in indeterminate TBSRTC categories. Thus, studies including comparisons between the subgroups of the AUS-N subcategory with SFM category via a nuclear scoring schema in larger study groups may be beneficial in this regard.

Since, ROM is similar in aspirates with both mild nuclear and architectural atypia regardless of the presence or absence of concomitant architectural atypia, this subgroup is considered AUS-nuclear subcategory in the TBSRTC 3rd edition [6]. AUS-N&A subcategory had the third highest ROM rate following the AUS-N1 subgroup and AUS-N subcategory in the current study. The rate of LRN was also high in the surgical materials of the AUS-N&A subcategory. Therefore, we examined the NS distribution of AUS-N&A subcategory. These evaluations showed that the AUS-N&A subcategory with NS3 features had significantly higher ROM (60.0%) than AUS-N&A aspirates evaluated as NS2 (28.1%) (*p* = 0.006). Also, ROM for extensive NS2 features (40.6%) was higher than the focal NS2 features (15.6%) with close to the significance (*p* = 0.052). These findings emphasize the importance of nuclear scoring in thyroid FNA samples. AUS-A subcategory and AUS-A2 subgroup significantly indicated BNs on histological examination. Considering the relationship of three-dimensional groups with high ROM, which is emphasized in some previous studies [31, 32] and in the current study, perhaps architectural atypia, as well as nuclear atypia, may be nature-defining. So, detailed definition of architectural features as well as nuclear scoring and raising awareness in terms of three-dimensional groups may be useful in the correct evaluation of thyroid cytology.

On the other hand, a significantly higher rate of NND was detected in the surgical specimens of nodules considered AUS-O and AUS-NOS subcategories with the lowest ROM value in the AUS-O category (5.6%) which was followed by AUS-NOS (11.5%). The ROM value determined for the AUS-O subcategory in this study is lower than the values reported in previous studies [16, 37–39] and the mean value reported in the TBSRTC 3rd edition [6]. A similar situation existed for the AUS-NOS subcategory, undoubtedly the category with the most different definitions [6, 16–19, 21, 31, 32, 39–42]. Park et al. [10, 31] used similar criteria for AUS-NOS as our study and detected a ROM of 14.5%, and this rate supports our study. Since clinical, radiological, and laboratory findings are important in cytological evaluations for the AUS-O and AUS-NOS subcategories, it may be due to the fact that evaluation of the study group in the multidisciplinary endocrine diseases council contributed to the differential diagnosis of these subcategories and to the selection of appropriate patients for surgery in the present study. Therefore, the integration of clinical and laboratory findings with cytological findings may save the AUS-NOS subcategory from being a wastebasket category.

In this study, all categories except the AUS-L subcategory were compared pairwise in terms of ROM. No statistically significant difference was observed between AUS-N and AUS-N&A subcategories in terms of ROM. In other words, these two subcategories exhibited similar patterns in terms of ROM. However, the ROM values of these two categories were found to be significantly higher than the ROM values of other subcategories. Therefore, these two subcategories were combined to form a group, and all other subcategories were combined to form a separate group. And finally, the ROM value of the group consisting of AUS-N and AUS-N&A subcategories was found to be significantly higher than the other group. Based on these results, the group consisting of AUS-N and AUS-N&A subcategories named as “high-risk group,” and the group consistent of other subcategories named as “low-risk group.” These last groups created actually correspond to the AUS-nuclear and AUS-other subcategories expressed in the TBSRTC 3rd edition [6]. Studies reported following the first introduction of TBSRTC [7] and a recently reported study based on TBSRTC 3rd edition [6], comparing the changes reported as AUS-nuclear or AUS-cytological atypia with other AUS defining changes, individually or in different combinations are summarized in Table 13 [8, 15–18, 20–23, 25, 26, 37–39, 43–48]. When the table is examined, it is obvious that the ROM values of the subcategories defined as AUS-nuclear atypia or AUS-cytological atypia are higher than other groups, both in individual studies and overall. Therefore, it can be predicted that the subcategorization proposed in the TBSRTC 3rd edition will have a positive impact on clinical practice. On the other hand, the majority of these studies belong to the era before NIFTP and malignancy rates included NIFTP. However, in the post-NIFTP period, NIFTP is included in the malignant category in some of these studies [37], while in others, the status of NIFTP is not fully explained [16, 26, 38, 47]. NIFTP was not even mentioned in several studies [8, 25, 48]. It is understood from this table that in studies that had not evaluate the NIFTP in the malignant category, ROM values for AUS-nuclear atypia or AUS-cytological atypia continue to be higher than other subcategories. Valderrabano et al. [49] reported that the ROM in cases with nuclear atypia was 46%, and the ROM in cases without nuclear atypia was 18% (NIFTP is considered in the malignant category) in a meta-analysis including 20 studies. In the current study, diagnostic categories were created based on the 5th edition (2022 Beta version) of the WHO Classification of Endocrine and Neuroendocrine Tumours [28], and NIFTP and other LRNs were examined as separate diagnostic categories. Therefore, the ROM values reported in the current study can be considered more objective. The current study had the advantage of comparing the TBSRTC 2nd edition [9] and TBSRTC 3rd edition [6] periods. Namely, the subcategories and subgroups included in this study offer the opportunity to compare in terms of ROM separately according to both TBSRTC editions. According to these data, based on the TBSRTC 2nd edition criteria for nuclear atypia, ROM was 48.2% [9], while it was 42.0% according to the TBSRTC 3rd edition [6]. In other words, when the nuclear and architectural atypia criteria are considered under the title of nuclear atypia, the ROM value actually decreases because the group dynamics are affected. Therefore, although thyroid cytology continues to be reported based on the criteria defined in the TBSRTC 3rd edition and the suggested subcategories, reporting the criteria defining the subcategory as a subgroup may contribute to the management of nodules. In addition, the use of nuclear scoring for the AUS-nuclear subcategory and the identification of detailed architectural patterns rather than general architectural atypia may have an impact on ROM values, and perhaps in the future, the nature of these two subcategories can be determined more realistically, and the subcategories can be included in other categories. Since the AUS-nuclear subcategory by definition also includes atypical cyst-lining cells and histiocytoid cells, this category actually includes not only nuclear atypia but also atypia partially due to cytoplasmic features. On the other hand, it also includes architectural atypia under its umbrella. For these reasons, perhaps defining the AUS-nuclear subcategory as “AUS-high risk group” and the AUS-other subcategory as “AUS-low risk group” can be discussed as a more appropriate terminological choice.

The present study has some limitations. First of all, this study is a retrospective study. Additionally, as discussed in the TBSRTC 3rd edition, the ROM value was calculated only for patients who underwent surgery; the actual ROM value in patients who did not undergo surgery is unknown. Since that, the rate for AUS and ROM for SFM in the current study were achieved from the records of previous FNAs, presented rates do not represent the rates likely to be achieved according to the criteria used in this study.

On the other hand, this study also has strengths. First of all, the number of cases with surgical follow-ups in the present study is higher than most of the previous studies except the study reported by Mathur et al. [39]. Also, the aspirates and surgical specimens of the cases were reevaluated and revised according to current diagnostic guidelines. Therefore, results and the definitions directly express current practice. Correlations between the aspirated nodule and the target lesion observed in the surgical specimen were made according to very strict criteria, and incompatible cases were excluded from the study. Since the cytomorphological features in recurrent aspirations may also include regenerative changes resulting from the previous aspirations, the initial aspirations of the nodules were included in the study in order to evaluate the real situation at the zero point, and repeated aspirations were excluded from the study. And finally, this study presents a review of previous studies investigating the ROM for subcategories of AUS following the first edition of TBSRTC (Table 13).

In conclusion, the accumulation of data reported in the literature since the TBSRTC 1st edition represented that the risk of malignancy in the subcategories defined as AUS-nuclear or AUS-cytological atypia is higher than other subcategories. Therefore, it can be predicted that the “AUS-nuclear” and “AUS-other” subcategorization proposed for the AUS category in the TBSRTC 3rd edition will be beneficial in the clinical management of thyroid nodules. However, considering the definitional framework of the AUS-nuclear subcategory, changing the nomenclature of AUS subcategories as “AUS-high risk” and “AUS-low risk” is open to debate. Since that, significant differences in terms of ROM were detected among the subgroups of AUS-nuclear atypia subcategory, the use of a nuclear scoring system in aspirates with AUS-nuclear atypia subcategory can convert nuclear atypia into numerical values and lead to a more objective evaluation. According to the results of our study, it can be predicted that subcategorization may not be the end point, and nuclear scoring with evaluation of architectural patterns according to strict criteria may provide data for remodeling of TBSRTC categories. In this regard, future studies that include the evaluation of nuclear atypia based on nuclear scoring in aspirates diagnosed as AUS may be beneficial and may guide the recommendations of the TBSRTC subsequent edition.

## Data Availability

All data analyzed during this study are included in this published article.
